# Molybdenum-isotope signals and cerium anomalies in Palaeoproterozoic manganese ore survive high-grade metamorphism

**DOI:** 10.1038/s41598-019-40998-5

**Published:** 2019-03-14

**Authors:** Alexandre Raphael Cabral, Armin Zeh, Nívea Cristina Vianna, Lukáš Ackerman, Jan Pašava, Bernd Lehmann, Vladislav Chrastný

**Affiliations:** 1 Centro de Pesquisa Professor Manoel Teixeira da Costa, Instituto de Geociências, Universidade Federal de Minas Gerais (UFMG), Belo Horizonte, MG 31270-901 Brazil; 2 Centro de Desenvolvimento da Tecnologia Nuclear (CDTN), Belo Horizonte, MG 31270-901 Brazil; 3 Institut für Angewandte Geowissenschaften, Mineralogie und Petrologie, Karlsruher Institut für Technologie (KIT), Adenauerring 20b, Geb. 50.40, 76131 Karlsruhe, Germany; 4 Vale Manganês S.A., Rua Duque de Caxias s/n, Morro da Mina, Conselheiro Lafaiete, MG 36401-195 Brazil; 5 Institute of Geology, The Czech Academy of Sciences, 165 00 Prague, Czech Republic; 6 Czech Geological Survey, Geologická 6, 152 00, Prague 5, Czech Republic; 7 Mineral Resources, Technical University of Clausthal, Clausthal-Zellerfeld, Adolph-Roemer-Str. 2a, 38678 Clausthal-Zellerfeld, Germany; 8 Department of Environmental Geosciences, Faculty of Environmental Sciences, Czech University of Life Sciences Prague, Kamýcká, 129,165 00 Prague, Czech Republic

## Abstract

Molybdenum (Mo) and its isotopes have been used to retrieve palaeoenvironmental information on the ocean–atmosphere system through geological time. Their application has so far been restricted to rocks least affected by severe metamorphism and deformation, which may erase or alter palaeoenvironmental signals. Environmental Mo-isotope signatures can be retrieved if the more manganese (Mn)-enriched rocks are isotopically depleted and the maximum range of δ^98^Mo values is close to the ~2.7‰ Mo-isotope fractionation known from Mo sorption onto Mn oxides at low temperature. Here, we show that the Morro da Mina Mn-ore deposit in Minas Gerais, Brazil, contains Mn-silicate–carbonate ore and associated graphitic schist that likely preserve δ^98^Mo of Palaeoproterozoic seawater, despite a metamorphic overprint of at least 600 °C. The extent of Mo-isotope fractionation between the Mn-silicate–carbonate ore and the graphitic schist is similar to modern Mn-oxide precipitates and seawater. Differences in δ^98^Mo signals are broadly reflected in cerium (Ce) anomalies, which suggest an oxic–anoxic-stratified Palaeoproterozoic ocean.

## Introduction

Assessing when and how oceans became oxygenated is crucial not only to trace the evolution of early life, but also to understand the genesis of metalliferous deposits in marine settings. Metals sensitive to variations in reduction–oxidation (redox) conditions have the potential to resolve the palaeoenvironmental setting in which marine sediments have been deposited^1–3^. Manganese and Mo represent such redox-sensitive metals. The former, soluble as divalent Mn, requires free oxygen in seawater to form Mn-oxide particles. The latter is dissolved as hexavalent Mo in seawater and accumulates in organic-matter-rich sediments such as black shales^1,4–6^, reflecting the Mo-isotope composition (δ^98^Mo) of seawater only at H_2_S concentrations that are sufficiently high for the quantitative conversion of molybdate to thiomolybdate, which is then trapped by organic matter in euxinic waters^7,8^. In contrast, substantial fractionation of −2.7‰ in δ^98^Mo is associated with the adsorption of Mo onto Mn-oxide particles^9^.

The application of Mo as a palaeoenvironmental proxy to black shales and other marine sediments has essentially been restricted to sequences that experienced incipient metamorphism and deformation^6,8,10–12^, as they may affect depositional signals. Nevertheless, recent study has indicated that even greenschist-facies metamorphism is unable to erase depositional δ^98^Mo signals^13^. This indication is built on a positive correlation between Fe/Mn ratios and Mo-isotope values obtained from the ~2.95-Ga-old Sinqeni iron formation of the Pongola Supergroup, South Africa. Depositional δ^98^Mo signals have also been retained in the ~1.85-Ga-old Stambaugh Formation of the Paint River, Michigan, USA, which was metamorphosed to greenschist facies^14^. However, it remains unclear whether such a correlation is universal and realistic for rocks metamorphosed at higher grade, such as those found at the Morro da Mina Mn-ore deposit in Minas Gerais, Brazil. At Morro da Mina, amphibolite-facies metamorphism of marine sediments rich in Mn and organic matter resulted in the formation of queluzite, a Mn-silicate–carbonate rock containing graphite–molybdenite (MoS_2_) intergrowths, and Mo-bearing graphitic schist^15^. Both rock types, queluzite and graphitic schist (Fig. S1), should record deposition in euxinic waters to account for Mo enrichment. Thus, Morro da Mina offers a remarkable opportunity to apply the Mo proxy to Mn ore that attained at least 600 °C, as well as to its associated graphitic schist. We report U–Pb ages of detrital zircon grains from (i) quartzite, and (ii) granodioritic dyke in the Mn ore, to bracket the depositional age of the Morro da Mina queluzite in the Barbacena greenstone belt. Furthermore, δ^13^C and δ^98^Mo results, in addition to major and trace elements, place new constraints on the palaeoenvironmental conditions of Mn deposition, and demonstrate that Mo-isotope signals can be retrieved after amphibolite-facies metamorphism.

**Figure 1:**
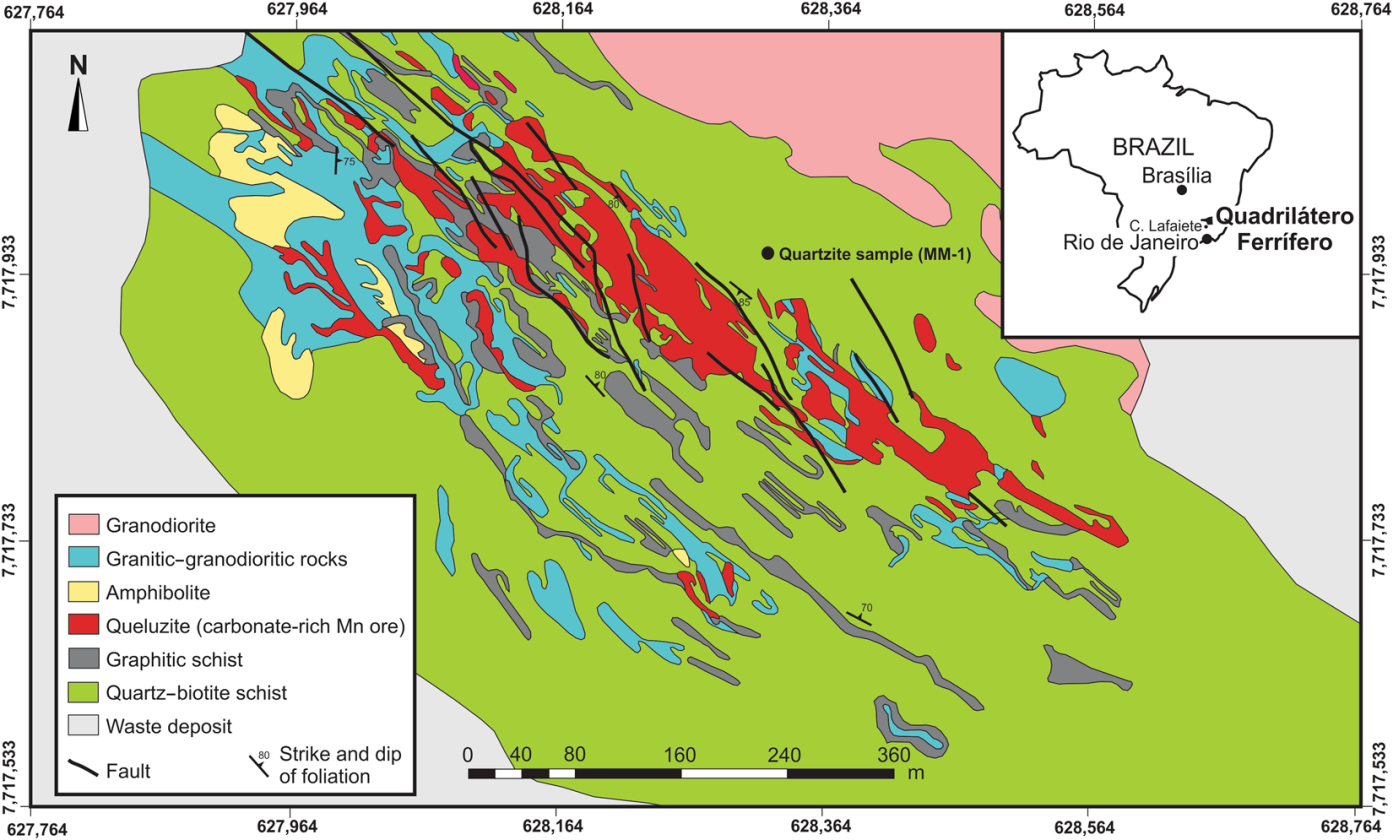
Geological overview of the Morro da Mina Mn-ore deposit (geology mapping by N.C.V.). For simplification, quartzite intercalations in quartz–biotite schist are not shown, but the location of the quartzite sample MM-1, from which detrital zircon grains were recovered (Fig. 2).

Morro da Mina is a Mn-ore deposit in operation since 1902, located to the south of the Quadrilátero Ferrífero of Minas Gerais, about 35 km southwest of Ouro Preto. Its main ore type is queluzite, which consists mainly of Mn carbonate accompanied by a variety of Mn-silicate minerals including spessartine [Mn^2+^_3_Al_2_(SiO_4_)_3_], rhodonite [Mn^2+^SiO_3_] and its polymorph pyroxmangite, tephroite [Mn_2_^+2^(SiO_4_)], and widespread dissemination of graphite and alabandite [MnS]^16–22^. Queluzite is a characteristic rock of the Lafaiete Formation, a Mn-rich unit that extends for over 100 km^23,24^, and forms part of the Barbacena greenstone belt^25^. The queluzite ore contains intercalations of quartz–biotite schist, graphitic schist, quartzite, garnet–amphibole schist and amphibolite (Fig. 1). This rock succession is isoclinally folded and sheared along ductile zones, which are marked by graphitic schist. Ductile shearing shaped queluzite into sigmoidal orebodies, of up 100 m in thickness. The folded succession is locally intruded by granodioritic dykes^16,21^. The assemblage rhodochrosite–tephroite–pyroxmangite indicates a minimum metamorphic temperature of 600 °C^26^, but even higher temperatures due to the thermal effect of the granodioritic dykes cannot be excluded. Until now, the ages of the sedimentary protolith of the Morro da Mina queluzite and the tectono-metamorphic overprint have been unclear.

**Figure 2:**
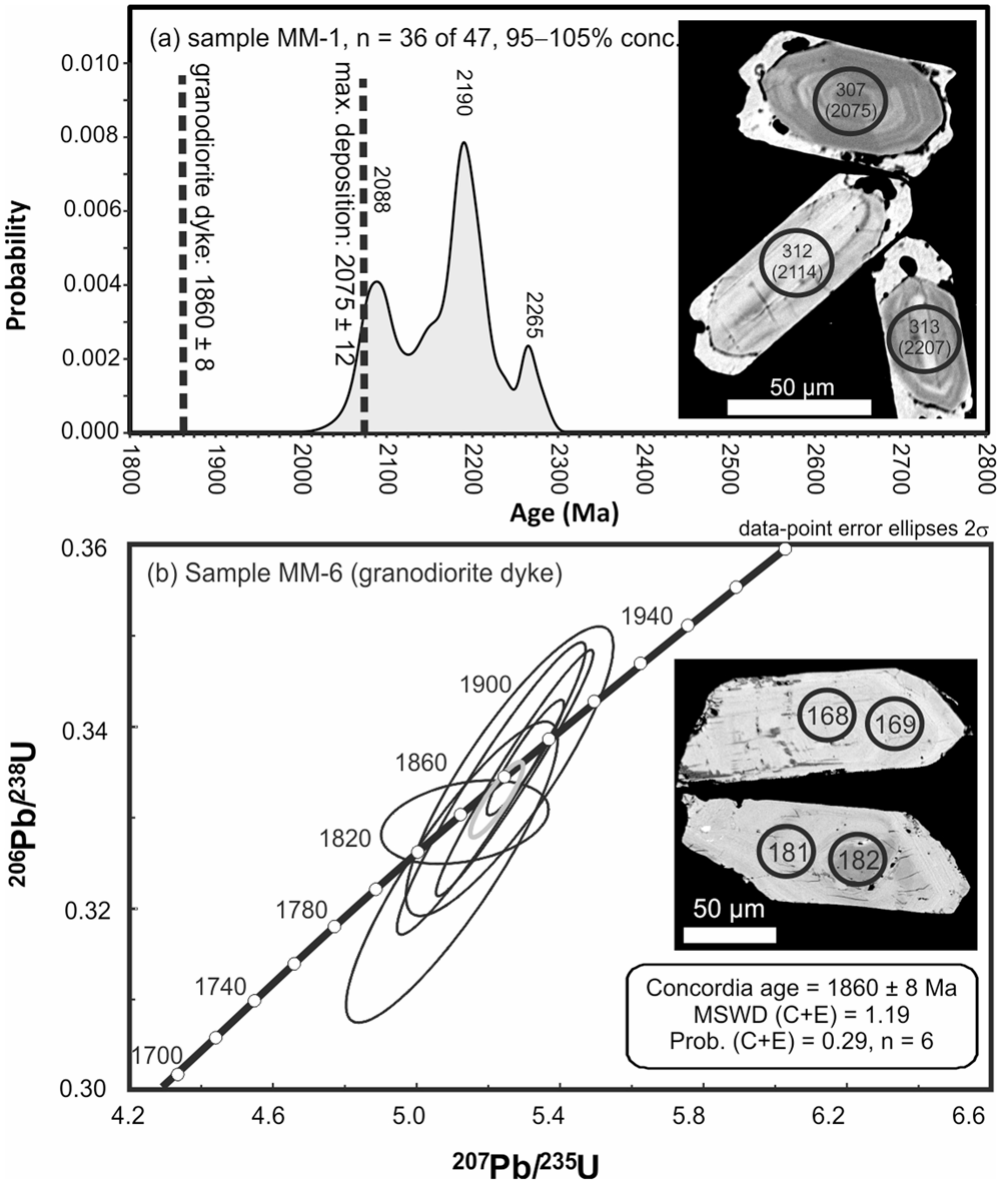
Evidence for a Palaeoproterozoic age for the Morro da Mina Mn-ore deposit. (**a**) Diagram of U–Pb age vs. probability for detrital grains of zircon from quartzite of the Mn-ore sequence at Morro da Mina. (**b**) Concordia diagram for magmatic zircon from a granodiorite dyke hosted in the Mn ore – i.e., queluzite. Insets in both diagrams show backscattered-electron images of zircon grains on which analytical spots are marked. Spot numbers refer to analyses presented in Table S1 (Supplementary Information). The deposition of manganiferous sediments is constrained between about 2.07 and 1.86 Ga.

Zircon grains were separated from a quartzite and a queluzite-hosted granodioritic dyke for U–Pb geochronology. Detrital zircon grains from the quartzite sample show cores with a typically magmatic growth zoning, generally surrounded by tiny outgrowths without any zoning (Fig. 2a, inset). The detrital zircon grains yielded Palaeoproterozoic ages, mostly at 2265 Ma, 2150 Ma and 2088 Ma (Table S1, Supplementary Information). The youngest detrital grain gave a concordant age of 2075 ± 12 Ma, which represents the maximum depositional age for the queluzite protolith (Fig. 2a). Zircon grains from the post-tectonic granodioritic dyke yielded a concordant age of 1860 ± 8 Ma (Fig. 2b). Collectively, the ages indicate that the deposition of Mn-rich sediments took place during the Palaeoproterozoic between 2.07 and 1.86 Ga (Fig. 2a).

**Figure 3:**
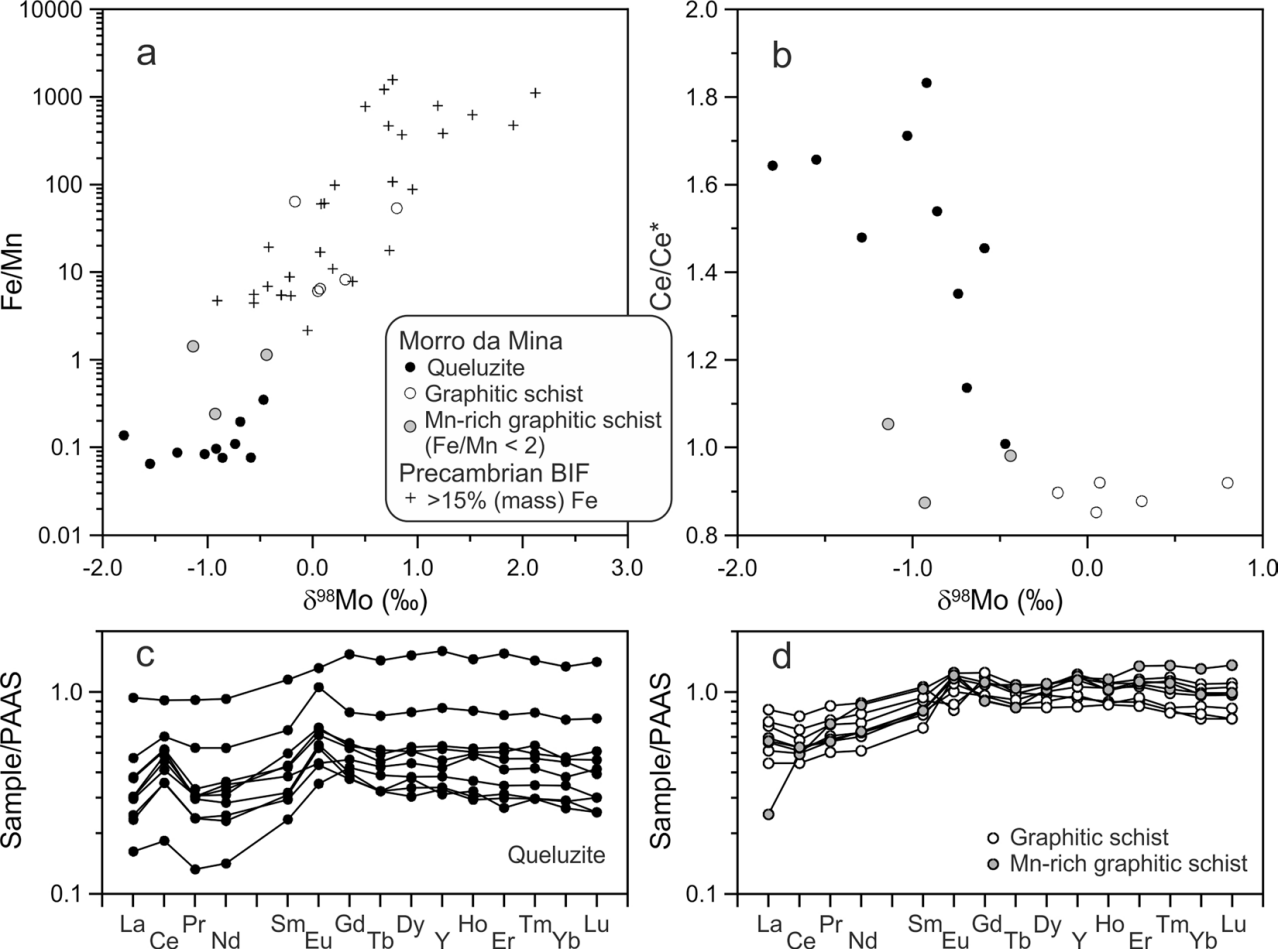
Palaeoenvironmental signals for queluzite and graphitic schist from the Morro da Mina Mn-ore deposit. (**a**) Positive correlation between δ^98^Mo values and whole-rock Fe/Mn ratios from Morro da Mina in comparison with Archaean and Palaeoproterozoic banded iron formations – BIF^13^. The data for Morro da Mina define the high-Mn end of the correlation, suggesting that the Mo-isotope signals of Mn oxidation (δ^98^Mo < 0) were transferred to reduced, sulfide-bearing sediments that gave rise to the queluzite Mn ore mined at Morro da Mina. (**b**) Molybdenum-isotope values are negatively correlated with Ce anomalies (Ce/Ce*, calculated using the equation of Lawrence and Kamber^41^; PAAS refers to Post-Archaean Australian Shale^42^). This correlation connects the Mo-isotope signals of Mn oxidation with Ce recycling due to reductive dissolution of Mn-oxide particles across the contact between oxic and euxinic waters – i.e., redoxcline. (**c**–**d**). The positive Ce anomaly of most queluzite samples is mirrored in the negative Ce anomaly of graphitic schist.

A sample collection of queluzite (n = 10) and graphitic schist (n = 9) was analysed for their whole-rock major- and trace-element contents, and Mo and C isotopic compositions (Supplementary Table S2). The queluzite samples represent the main Mn-ore type at Morro da Mina. Their Mn contents are between ~15 and 33% (mass), with Fe/Mn ratios varying from 0.06 and 0.35. The samples of graphitic schist exemplify ductile shear zones poorer in Mn (Fe/Mn > 2), but with locally elevated Mn concentrations (Fe/Mn < 2 − i.e., Mn-rich graphitic schist). The queluzite and graphite schist significantly differ in their δ^98^Mo values, between −1.80 and −0.47‰, and between −0.17 and 0.80‰, respectively (Fig. 3a). The Mn-rich graphitic schist has δ^98^Mo values between −1.44 and −0.44‰, which are essentially within the queluzite δ^98^Mo range. The maximum difference in δ^98^Mo is 2.6‰ (Δ^98^Mo), which is close to that between modern seawater and seafloor Mn-oxide crusts and nodules (~3.0‰ δ^98^Mo^27,28^). The difference of 2.6‰ δ^98^Mo also agrees with the experimentally determined δ^98^Mo fractionation through Mo adsorption onto Mn-oxide particles^9^. The δ^98^Mo values and Fe/Mn ratios for both queluzite and graphitic schist follow the same positive correlation trend, which was previously delineated by Planavsky *et al*. (ref.^13^) for the Archaean Sinqeni iron formation in South Africa. Those authors interpreted this trend to reflect inheritance of the δ^98^Mo signal originally acquired during Mn oxidation (Fig. 3a). By analogy, this interpretation is extended to Morro da Mina despite tectonic and metamorphic overprint.

Nevertheless, the depositional setting at Morro da Mina differs from that envisaged for the extremely sulfide-poor Archaean Sinqeni iron formation in South Africa, which likely captured its Mo-isotope signals from Mn-oxide particles during their reductive dissolution below the seawater–sediment interface^13^. Conversely, the reductive dissolution of Mn-oxide particles possibly occurred above the sediment layer within a redox-stratified ocean at Morro da Mina. In such an ocean, Mn-oxide particles that had scavenged Mo from oxic shallow waters became dissolved below a redoxcline, in bottom waters rich in H_2_S and organic matter. This interpretation is compatible with the ubiquitous dissemination of alabandite and the omnipresence of graphite in the Morro da Mina queluzite, as well as with the finding of high tungsten (W) contents in molybdenite^15^. Values of δ^13^C for the queluzite and graphitic schist are respectively between −20.8 and −14.5‰, and between −22.5 and −20.8‰ (Supplementary Table S2), suggesting a largely organic origin for C in both graphite and carbonate (e.g., ref.^29^). There is a positive correlation between δ^13^C and the mass ratio of carbonate C to total C (Fig. S2a), which allows to identify the δ^13^C composition of the carbonate component at about −12‰ (Fig. S2b), in agreement with 6 selective measurements of the δ^13^C composition of carbonate, which gave an average of −11 ± 2‰. These data suggest an origin via remineralisation by pore fluids. While the dissolved load of divalent Mn was fixed in sediments as Mn carbonate and sulfide (alabandite), released molybdate ions with inherited δ^98^Mo from Mn-oxide particles above the redoxcline were trapped by S-rich organic matter in euxinic bottom waters^7,30^. The latter process is expressed as graphite–molybdenite intergrowths in the Morro da Mina queluzite^15^. It should be mentioned that a shuttle of Mn oxides to the seawater–sediment interface cannot be excluded, but the scenario of Mn redox cycling across a redoxcline appears more compelling if Ce is considered.

Cerium is a key element because of its ability to accumulate in seafloor Mn-oxide deposits as Ce(IV), leading to the mirrored Ce depletion in modern seawater^31,32^. The Morro da Mina queluzite has positive Ce anomalies (up to 1.8), which indicate mediation by particulate Mn-oxide formed above the redoxcline. On the other hand, the graphitic schist has slightly negative Ce anomalies, which is an indication of precipitation from Ce-depleted seawater above the redoxcline. Therefore, the different Ce anomalies provide evidence that Mn redox cycling took place across an oxic–euxinic water interface. This interpretation is supported by the negative trend between Ce anomaly and δ^98^Mo (Fig. 3b–d), which is complementary to the positive correlation of δ^98^Mo vs. Fe/Mn (Fig. 3a), reflecting the amount of particulate Mn oxide during sediment deposition. Such a scenario of Mn-oxide shuttle to euxinic bottom waters would also have led to strong enrichment in authigenic Mo in sediments, but little or no authigenic enrichment in U^33^. In this context, we note that the Morro da Mina queluzite has enrichment factors between 30 and 500 for Mo, but only between 0.4 and 1.5 for U, compared to the average composition of the upper continental crust.

**Figure 4:**
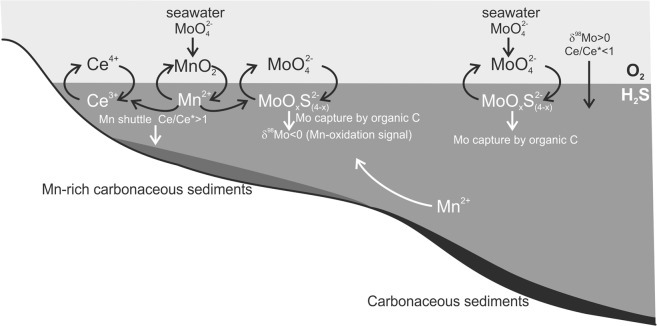
Conceptual depositional model for manganiferous sediments at Morro da Mina. Left side – Mn shuttle in a redox-stratified ocean: Mo (with δ^98^Mo<0) and Ce were preferentially adsorbed onto Mn-oxide particles in oxidized seawater above the redoxcline. Across the redoxcline Mn-oxide particles underwent reductive dissolution, leading to the formation of Mn-carbonate particles. Molybdenum (with δ^98^Mo<0), released by the reductive dissolution, was scavenged by organic C in euxinic seawater and transferred to Mn-rich carbonaceous sediments. The reductive dissolution of Mn-oxide particles across the redoxcline also caused Ce enrichment (Ce/Ce* >1). Right side: in a deeper part of the basin, where bottom waters were depleted in Mn due to Mn upwelling to swallow waters, seawater Mo was directly scavenged by organic C and deposited as carbonaceous muds that captured the Mo-isotope signal of Ce-depleted seawater (δ^98^Mo>0, Ce/Ce* <1). The Mn-rich carbonaceous sediments were metamorphosed at high temperature (>600 °C) to queluzite, and the carbonaceous sediments to graphitic schist. The former is a Mn-carbonate rock containing Mn silicates, Mn sulfide and graphite; the latter is poorer in Mn and marks ductile shear zones. Despite metamorphic and tectonic overprints, palaeoenvironmental information – i.e., primary differences in Mo-isotope signals – is still preserved and can be retrieved.

Placing detailed constraints on the palaeoenvironment at Morro da Mina is problematic due to: (i) the tectonic overprint, which obliterated the metasedimentary stratigraphy; (ii) the substantial clastic component, reflected in the contents of Al_2_O_3_ >5% (Supplementary Table S2), which obscures the seawater signature. Because of the tectonic overprint, it is not possible to ascertain how the queluzite and graphitic schist are stratigraphically related to each other, but both rocks were originally sediments deposited under euxinic conditions. It is then possible to advance further interpretations on their Mo-isotope signals and rare-earth-element (REE) patterns. Most queluzite samples have positive Ce anomalies, which can be explained by the reductive dissolution of Mn-oxide particles, from which Ce was released and delivered to Mn-rich sediments (see above). The positive anomalies of Eu likely reflect a seawater signature from its interaction with hot basalt – i.e., high-temperature alteration of basalt, from which Eu is leached by hydrothermal fluids venting into seawater^34^. Upwelling of Mn-rich bottom waters would carry this Eu signature. Sedimentation of organic-matter-rich mud took place in a deeper part of the basin, having captured the signal of seawater depleted in Ce, but enriched in the heavy Mo isotope (δ^98^Mo > 0), which are characteristics of Mn-oxide-particle formation in oxic waters. Our particle-shuttle scenario is schematically depicted in Fig. 4, a modern analogue of which is the Baltic Sea, where Mn carbonate and Mn sulfide account for total Mn contents of up to 32% (mass)^35^, equivalent to the Mn-ore grade of Morro da Mina. The black-shale precursor of the graphitic schist represents, with respect to Mo, Palaeoproterozoic seawater with positive δ^98^Mo (cf. ref.^8^), and its essentially flat REE patterns denote clastic input. In summary, the highly metamorphosed Mn-rich rocks at Morro da Mina appear to retain depositional Mo-isotope signatures. However, seawater Mo-isotope values can vary and the lightest and heaviest values are not constrained, but assumed, to be the same age. Although such uncertainties may have influenced the fractionation of Mo isotopes, it is remarkable that the difference between the two end-member δ^98^Mo values is not only similar to that reported from sites where bedding and other sedimentary features are commonly well preserved^12,14^, but also close to the experimentally predicted isotopic fractionation of Mo onto Mn oxides at low temperature^9^. At this point, it is worth mentioning that metamorphic, high-temperature fractionations of Mo isotopes would have been so small that their effect on the predicted low-temperature Δ^98^Mo is not perceptible.

One important implication refers to the absence of sulfide and ferrous Fe in the water column beneath “local marine oxygen oases” of photosynthetic microorganisms, as a requirement for Mn-oxide-bound Mo to be shuttled to sediments^13^. Our study indicates that the Mo-isotope signal of Mn oxidation can be retained in euxinic waters below the Mn redoxcline. The aforementioned oxygen oases were not large enough to have formed a Mn redoxcline in Archaean banded-iron-formation (BIF) settings to generate the combined signals of Ce anomalies and Mo-isotope fractionations, as recorded here (Fig. 3b), given the general lack of positive Ce anomalies in Archaean BIF^36^. Archaean oxygen oases may nevertheless have expanded to a Mn redoxcline forming combined Ce–Mo signals, perhaps in the ca. 3.1-Ga-old Iron Ore Group, which hosts Mn deposits in the Singhbhum craton, eastern India^37^. Therefore, such Mn deposits in India and elsewhere have the potential of providing palaeoenvironmental information irrespective of metamorphic grade and tectonic overprint. The recognition that the Mo proxy may be retained in rocks containing graphite opens new perspectives of investigating Precambrian terranes that have otherwise been considered unsuitable for palaeoenvironmental studies because of metamorphism and deformation. This point is stressed here with respect to the depositional age for the queluzite sedimentary protolith (~2.07–1.86 Ga), which connects the Morro da Mina Mn deposit to the Lomagundi event^38^, when extensive removal of organic carbon from seawater was counterbalanced by a high rate of oxygen production^39^. This connection is further reinforced by the association of Mn deposits with black shales^40^, known in Palaeoproterozoic metamorphic terranes of West Africa, Gabon, India and northern Brazil^37^. The preservation of environmental signals – i.e., Mo-isotope fractionations and Ce anomalies – and their connections to such an event can thus be retrieved from medium- to high-grade metamorphic terranes.

## Methods

Methods are available as electronic Supplementary Information.

## Supplementary information

Supplementary Information
