# Supplementary material for: Molybdenum-isotope signals and cerium anomalies in Palaeoproterozoic manganese ore survive high-grade metamorphism
Source: Sci Rep. 2019 Mar 14;9:4570. doi: 10.1038/s41598-019-40998-5 (PMC6418314; doi:10.1038/s41598-019-40998-5)
Supplement: Supplementary file 1 — Supplementary Information [file 41598_2019_40998_MOESM1_ESM.docx]

**Supplementary Information**

**Molybdenum-isotope signals and cerium anomalies in Palaeoproterozoic manganese ore survive high-grade metamorphism**

**Alexandre Raphael Cabral^1,2^, Armin Zeh^3^, Nívea Cristina Vianna^4^, Lukáš Ackerman^5,6^, Jan Pašava^6^, Bernd Lehmann^7^ and Vladislav Chrastný^8^**

^1^ Centro de Pesquisa Professor Manoel Teixeira da Costa, Instituto de Geociências, Universidade Federal de Minas Gerais (UFMG), Belo Horizonte, MG, 31270-901, Brazil (arcab@ufmg.br)

^2^ Centro de Desenvolvimento da Tecnologia Nuclear (CDTN), Belo Horizonte, MG, 31270-901, Brazil

^3^ Institut für Angewandte Geowissenschaften, Mineralogie und Petrologie, Karlsruher Institut für Technologie (KIT), Adenauerring 20b, Geb. 50.40, 76131 Karlsruhe, Germany (armin.zeh@kit.edu)

^4^ Vale Manganês S.A., Rua Duque de Caxias s/n, Morro da Mina, Conselheiro Lafaiete, MG, 36.401-195, Brazil (nivea.cristina.viana@vale.com)

^5^ Institute of Geology, The Czech Academy of Sciences, 165 00 Prague, Czech Republic (ackerman@gli.cas.cz)

^6^ Czech Geological Survey, Geologická 6, 152 00 Prague 5, Czech Republic (jan.pasava@geology.cz)

^7^ Mineral Resources, Technical University of Clausthal, Clausthal-Zellerfeld, Adolph-Roemer-Str. 2a, 38678 Clausthal-Zellerfeld, Germany (bernd.lehmann@tu-clausthal.de)

^8^ Department of Environmental Geosciences, Faculty of Environmental Sciences, Czech University of Life Sciences Prague, Kamýcká 129,165 00 Prague, Czech Republic (chrastny@fzp.czu.cz)

**Metasedimentary rocks**

Two types of metasedimentary rocks were sampled for this study. One is queluzite, which is currently mined for Mn at Morro da Mina. The rock queluzite has about 30 % (mass) of total Mn^1^. It is essentially a Mn-silicate-carbonate rock, the Mn-carbonate component of which is characteristic and comprises rhodochrosite and manganoan calcite. The rock contains variable proportions of silicate minerals, but spessartine is the most abundant. Graphite and sulfide minerals, such as alabandite, pyrrhotite and pentlandite, are disseminated (Fig. S1a).

The other rock sampled is graphitic schist. Its prominent foliation is defined by the planar arrangement of graphite (Fig. S1b). The graphitic schist represents carbonaceous black shale that was highly deformed and metamorphosed under amphibolites-facies conditions.

**Methods**

*U–Pb dating of zircon*

Measurements for U, Th and Pb isotopes were performed on zircon grains mounted in epoxy, ground to expose their cores and then polished, using a ThermoScientific Element 2 sector field (SF) ICP-MS and a Resolution M-50 (Resonetics) 193-nm ArF excimer laser (ComPexPro 102F, Coherent), at the Goethe-University Frankfurt. Analytical procedures are described in Ref. 2 (and references therein). Data were acquired with a 20-second background measurement, followed by 21-second sample ablation. Laser spot size was 30 µm in diameter for unknowns and for reference zircon GJ-1 (primary standard), Plešovice and OG1. Ablation was performed in a He stream (~0.6 l min^-1^), which was mixed directly after the ablation cell with N_2_ (~0.6 ml min^-1^) and Ar (0.79 l min^-1^), prior to introduction into the Ar plasma of the SF-ICP-MS. Signal was tuned for maximum sensitivity for Pb and U while keeping oxide production, monitored as ^254^UO/^238^U, below 0.5–0.3%. Sensitivity achieved was in the range of 9000–14000 cps/µg g^-1^ for ^238^U with a 30-µm spot size, at 5.5 Hz and ~3.0 J cm^-2^ laser energy. Penetration depth was typically ~15 µm. Raw data were corrected offline for background signal, common Pb, laser-induced elemental fractionation, instrumental mass discrimination, and time-dependent elemental fractionation of Pb/U using an in-house MS Excel^©^ spread­sheet program^3,4^. A common-Pb correction was applied, based on the interference- and background-corrected ^204^Pb signal and a model Pb composition^5^. For the samples, calculated common ^206^Pb contents were mostly <0.5% of the total ^206^Pb. Laser-induced elemental fractionation and instrumental mass discrimination were corrected by normalization to the reference zircon GJ-1 (Ref. 6), as well as inter-elemental fractionation (^206^Pb*/^238^U) during the sample ablation. Drift during the analytical sessions was corrected and varied between 1 and 3%. Reported uncertainties (2σ) were propagated by quadratic addition of the external reproducibility (2 SD, standard deviation) obtained from the reference zircon GJ-1 and the within-run precision of each analysis (2 SE, standard error).

For ^207^Pb/^206^Pb, we used a ^207^Pb-signal dependent uncertainty propagation^4^. The ^207^Pb/^235^U ratio is derived from the normalised and error-propagated ^207^Pb/^206^Pb* and ^206^Pb*/^238^U ratios, assuming a ^238^U/^235^U natural abundance ratio of 137.88 and the uncertainty derived by quadratic addition of the propagated uncertainties of both ratios. Accuracy was verified by analyses of three reference materials of zircon – GJ-1, Plešovice and OG1. They respectively gave Concordia ages of 604 ± 1 Ma (n=26), 339 ± 1 Ma (n=26), and 3463 ± 6 Ma (n=11), in agreement with the data published in Ref. 7–8, and in-house TIMs results. Data were plotted using the software ISOPLOT^9^. Finally, results are presented in Table S1.

*Whole-rock chemical analysis*

Rock samples were ground in an agate mill. Major- and trace-element concentrations were determined by a multi-element package at Bureau Veritas Commodities Canada Ltd., Vancouver. The package combined ICP–OES and ICP–MS techniques, aqua-regia digestion and lithium-borate fusion. Total organic carbon (TOC) and total sulfur (TS) contents were measured

by a Leco infrared carbon–sulfur analyzer, while organic carbon was quantified after carbonate decomposition by phosphoric acid. Results are reported in Table S2.

*Carbon isotopic analysis*

Inorganic carbon was removed before analysis by acid washing with HCl followed by rinsing with water, drying at 60 °C and homogenisation. Measurements for C isotopes were performed at the Czech Geological Survey, Prague, by flash combustion in Fisons 1108 elemental analyzer, coupled with isotope-ratio mass spectrometer Delta V Advantage (ThermoFisher, Bremen, Germany), in continuous flow regime. Sample size was adjusted to contain a sufficient amount of C. Results are reported as δ^13^C values (in per mille, ‰), relative to V-PDB (Table S2). The international standard NBS 22 was used as reference material. Long-term reproducibility is better than 0.3‰ (2 SD).

Sample aliquots were selectively analysed for C isotopes of the carbonate component, using H_3_PO_4_ at 25 °C for 24 hours and the fractionation factor for MnCO^3^ according to Ref. 10. Results are reported as δ^13^C values (‰), relative to V-PDB (Table S2).

*Molybdenum isotopic analysis*

Molybdenum isotopic composition was determined in the joint laboratory of the Institute of Geology of the Czech Academy of Sciences (chemistry) and the Czech Geological Survey (mass spectrometry), using the double-spike technique and MC–ICP–MS. The ^97^Mo–^100^Mo double spike was prepared in a similar manner as described elsewhere (ref. 11,12). In brief, weighted aliquots (5–10 mg) of ^97^Mo and ^100^Mo metals (Oak Ridge National Laboratory, USA) were decomposed in Savillex PFA beakers using 14 M HNO_3_. After that, the solution was dried down, re-dissolved in diluted HNO_3_ with traces of HF (0.4 M HNO_3_–0.05 M HF), and double spike stock solution with concentration of 98.5 ppm was prepared with ^100^Mo/^97^Mo ratio of ~1.02.

Before sample decomposition, about 200 mg of rock material was incinerated at 600 °C for 10 hours to breakdown organic compounds. Subsequently, ~30 to 100 mg of incinerated sample was weighted into 15 ml Savillex beakers along with known amount of ^97^Mo–^100^Mo spike, and dissolved in a mixture of HF and HNO_3_ at 140 °C for 72 hours. Subsequently, the solution was evaporated to dryness and its residue was treated several times by 2 ml of 10 M HCl to ensure complete sample dissolution. Final residue was re-dissolved in 6 M HCl, dried again, re-dissolved in a mixture of 3 M HCl and 6 M HCl, followed by an addition of ascorbic acid to ensure reduction of Fe^3+^ to Fe^2+^ in remaining solution^13^, which was then ready for column chemistry. Molybdenum was separated from the matrix by ion-exchange chromatography using AG 1x8 resin (100–200 mesh, Eichrom) and 10 ml PP Biorad columns, and elution protocol described in detail^13^, with Mo collection using 12 ml of 1 M HCl. The Mo cut was dried down and re-dissolved two times by 100 µl of 14 M HNO_3_ and H_2_O_2_ to completely remove any possible organics. The final solution ready for mass-spectrometry measurement was prepared using 1 ml of 0.4 M HNO_3_ – 0.05 M HF. Total blank of the procedure, between 5 and 10 pg, was less than 1 % of total Mo content in samples and, therefore, was insignificant.

Molybdenum isotopic analysis was carried out using a multi-collector–inductively coupled plasma–mass spectrometer (MC–ICP–MS) instrument, Neptune (Thermo), equipped with nine Faraday detectors at the Czech Geological Survey. Samples were introduced into the Ar plasma via nebulizer Aridus II (Cetac, USA). The instrument was operated in a low-resolution mode, with typical sensitivity between ~3 and 4 V on ^96^Mo for a 300-ppb Mo solution using 50 µl nebulizer uptake. Background, always lower than 2 mV for all Mo masses, was negligible with no effect on the analyses. Between individual runs, the machine was washed out with a blank solution of of 0.4 M HNO_3_-0.05 M HF. Molybdenum isotopes of masses 94 (L2), 95 (L1), 96 (C), 98 (H2) and 100 (H4) were simultaneously detected while possible isobaric interferences from Ru and Zr were monitored through simultaneous collection of ^99^Ru (H3) and ^90^Zr (L4). Mass-bias correction was applied using a ^97^Mo–^100^Mo double spike. Optimum sample to spike ratio was found to be 1:1 to reach the condition of at least 95% natural isotopes coming from the sample, and 95% spiked isotopes coming from the double spike. Double-spike subtraction was made by iteration procedure using five blocks of cascade iteration. Isotopic compositions of Mo are reported as δ^98^Mo against NIST 3134 standard solution (Table S2). Total combined uncertainty (2SD) was 0.06 ‰ for the whole protocol, based on long-term reproducibility of δ^98^Mo values on double-spike standard mixtures and periodic analyses of several reference materials – e.g., BHVO-2, SGR-1b. During the course of this study, SGR-1b reference material (black shale, USGS) yielded δ^98^Mo values of 0.45 ± 0.03 ‰ (n = 9), in excellent agreement with previous studies^14,15^.

**References**

1. Viana, N.C. da S. Mineralogia, calcinação e nova classificação tipológica de minérios de manganês sílico-carbonatados. M.Sc. thesis, Universidade Federal de Ouro Preto, Ouro

Preto, Brazil (2009), http://www.repositorio.ufop.br/handle/123456789/3086.

1. Zeh, A. & Gerdes, A. U-Pb and Hf isotope record of detrital zircons from gold-bearing sediments of the Pietersburg Greenstone Belt (South Africa) – Is there a common provenance with the Witwatersrand Basin? *Precambri. Res.* **204–205**, 46–56 (2012).
2. Gerdes, A. & Zeh, A. Combined U-Pb and Hf isotope LA-(MC)ICP-MS analyses of detrital zircons: Comparison with SHRIMP and new constraints for the provenance and age of an Armorican metasediment in Central Germany. *Earth Planet. Sci. Lett.* **249**, 47–61 (2006).
3. Gerdes, A. & Zeh, A. Zircon formation *versus* zircon alteration – new insights from combined U-Pb and Lu-Hf *in-situ* LA-ICP-MS analyses, and consequences for the interpretation of Archean zircon from the Central Zone of the Limpopo Belt. *Chem. Geol.* **261**, 230–243 (2009).
4. Stacey J. S. & Kramers J. D. Approximation of terrestrial lead isotope evolution by a two-stage model. *Earth Planet. Sci. Lett.* **26**, 207–221 (1975).
5. Jackson, S.E., Pearson, N.J., Griffin, W.L. & Belousova, E.A.. The application of laser ablation-inductively coupled plasma-mass spectrometry to in situ U–Pb zircon geochronology. *Chem. Geol.* **211**, 47–69 (2004).
6. Sláma, J. et al.. Plešovice zircon – a new natural reference material for U-Pb and Hf isotope microanalysis. *Chem. Geol.* **249**, 1–35 (2008).
7. Stern, R.A., Bodorkos, S., Kamo, S.L., Hickman, A.H. & Corfu, F. Measurement of SIMS instrumental mass fractionation of Pb isotopes during zircon dating. *Geostandards and Geoanalytical Research* **33**,145–168 (2009).
8. Ludwig, K. Isoplot/Ex, rev. 2.49. A Geochronological Toolkit for Microsoft Excel. Berkeley Geochronology Center, Special Publication No 1a (2001).
9. Friedman, I. & O’Neil, J.R. Compilation of stable isotope fractionation factors of geochemical interest. U.S. Geol. Survey Prof. Paper, 440-KK (1977).
10. Siebert, C., Nagler, thomas F. & Kramers, J. D. Determination of Mo isotope fractionation by double-spike multicollector inductively coupled plasma mass spectrometry. *Geochemistry Geophys. Geosystems* **2,** 2000GC000124 (2001).
11. Skierszkan, E. K., Amini, M. & Weis, D. A practical guide for the design and implementation of the double-spike technique for precise determination of molybdenum isotope compositions of environmental samples. *Anal. Bioanal. Chem.* **407,** 1925–1935 (2015).
12. Willbold, M. *et al.* High-Precision Mass-Dependent Molybdenum Isotope Variations in Magmatic Rocks Determined by Double-Spike MC-ICP-MS. *Geostand. Geoanalytical Res.* **40,** 389–403 (2016).
13. Zhao, P. P. *et al.* Molybdenum Mass Fractions and Isotopic Compositions of International Geological Reference Materials. *Geostand. Geoanalytical Res.* **40,** 217–226 (2016).
14. Li, J., Zhu, X. kun, Tang, S. han & Zhang, K. High-Precision Measurement of Molybdenum Isotopic Compositions of Selected Geochemical Reference Materials. *Geostand. Geoanalytical Res.* **40,** 405–415 (2016).

Supplementary Figure 1
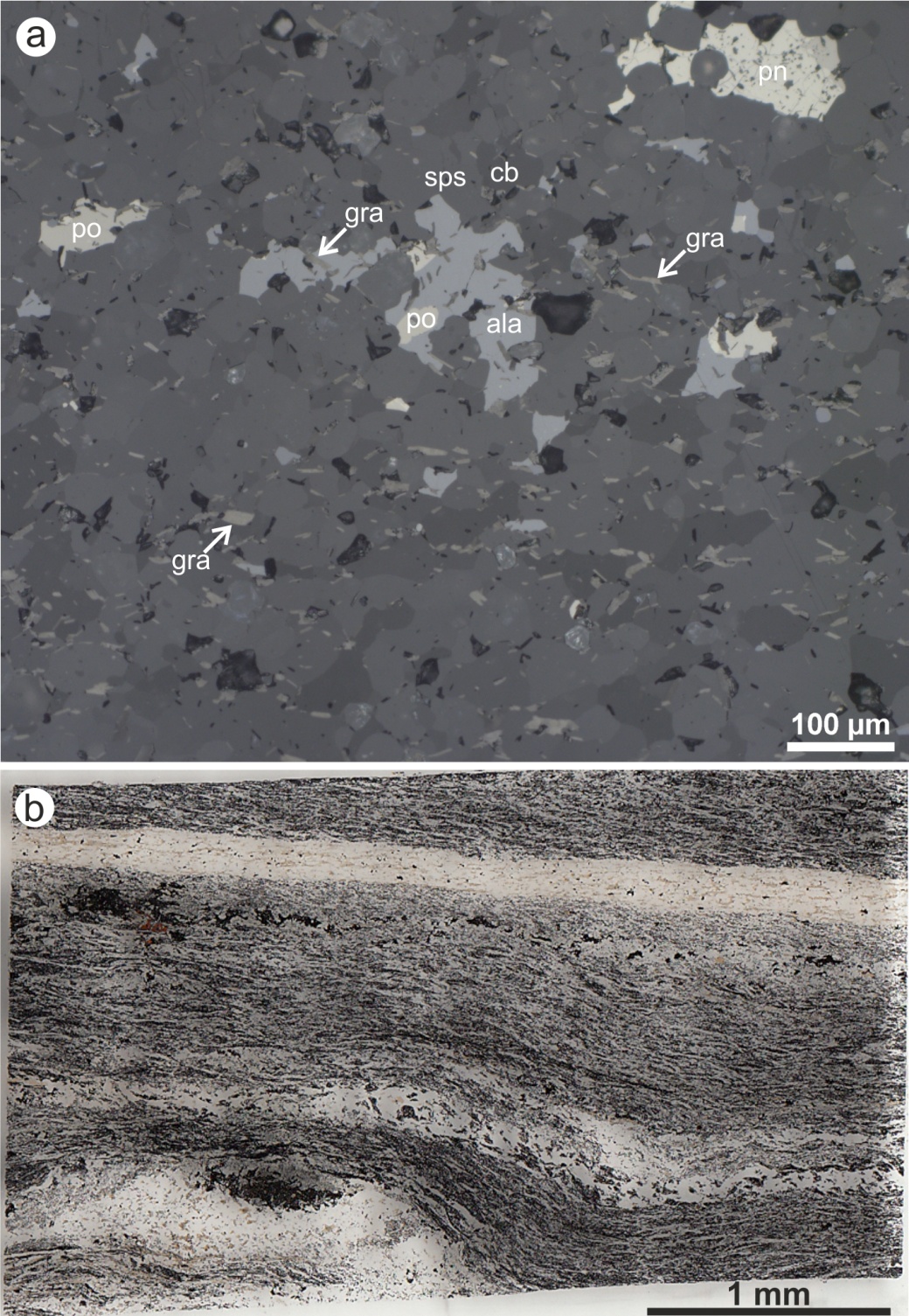


Fig. S1. a. Reflected-light photomicrograph of queluzite (sample MM-9), showing disseminated graphite (gra, arrowed), alabandite (ala), pyrrhotite (po) and pentlandite (pn), in a matrix of spessartine (sps) and Mn carbonate (cb). b. Photograph of a polished thin section of graphitic schist (sample MM-20). Its prominent foliation is defined by graphite (black). Sulfide minerals, pyrrhotite, chalcopyrite and sphalerite (undistinguished from abundant graphite), occurs as disseminations and pockets.

Supplementary Figure 2


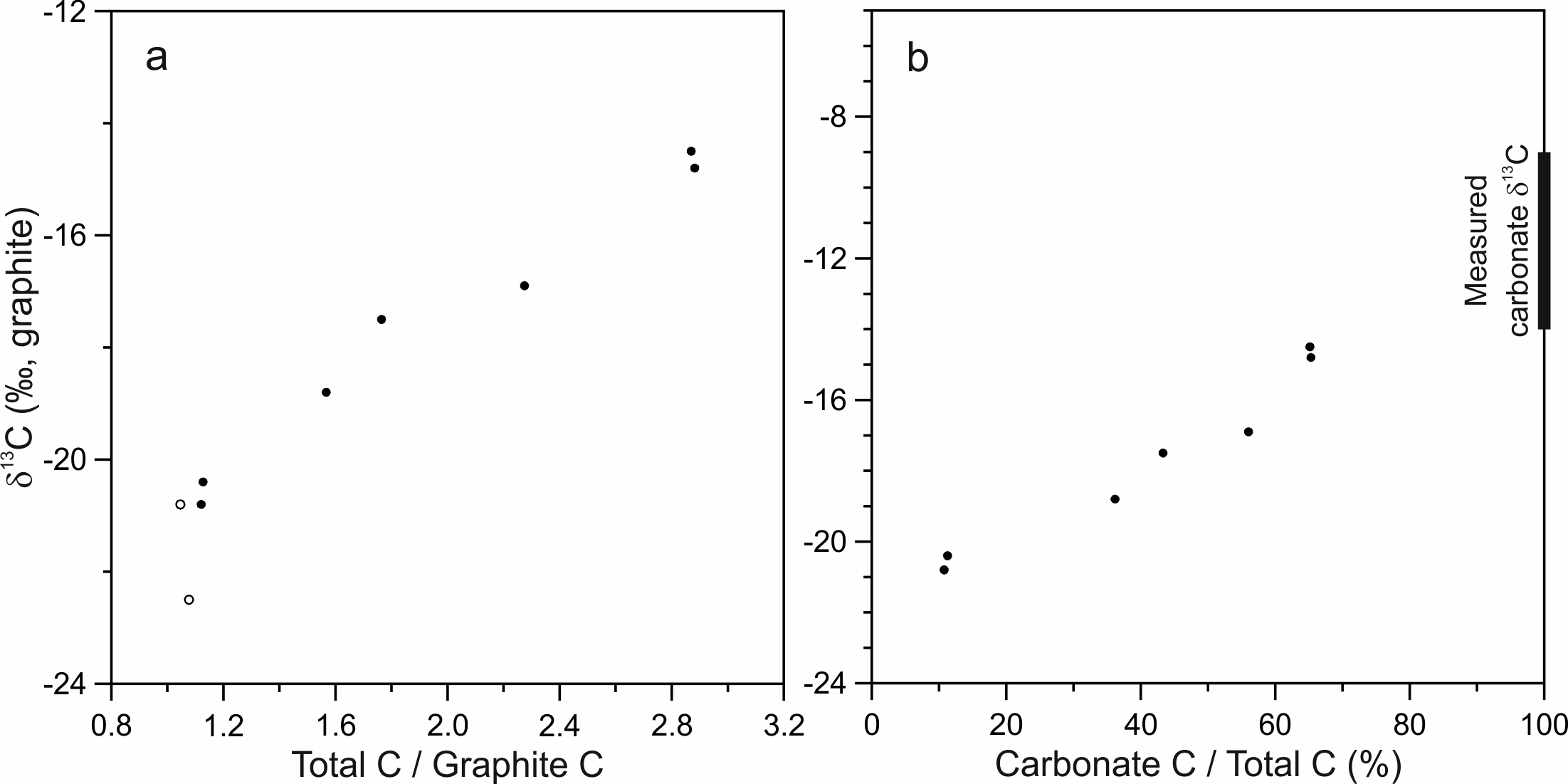


Fig. S2. a. Plot of ratio total C / graphite C vs. graphite-C-isotope composition for queluzite (filled circles) and graphitic schist (open circles) – data from Table S2. b. The positive linear correlation shown in a is used to estimate the C-isotope composition of carbonate from whole-rock chemical data (Table S2). The C-isotope composition is -11.5 ‰ δ^13^C at 100% carbonate, as estimated by linear regression. Black bar represents measured carbonate-δ^13^C values from selective analyses of whole-rock samples.

| Supplementary Table S1. Results of U–Pb dating | | | | | | | |  |  |  |  |  |  |  |  |  |  |  |  |  |  |
| --- | --- | --- | --- | --- | --- | --- | --- | --- | --- | --- | --- | --- | --- | --- | --- | --- | --- | --- | --- | --- | --- |
| grain |  | ^207^Pb^a^ | U^b^ | Pb^b^ | Th^b^ | ^206^Pbc^c^ | ^206^Pb^d^ | ±2σ | ^207^Pb^d^ | ±2σ | ^207^Pb^d^ | ±2σ | rho^e^ | ^206^Pb | ±2σ | ^207^Pb | ±2σ | ^207^Pb | ±2σ | conc.^f^ |  |
|  |  | (cps) | (ppm) | (ppm) | U | (%) | ^238^U | (%) | ^235^U | (%) | ^206^Pb | (%) |  | ^238^U | (Ma) | ^235^U | (Ma) | ^206^Pb | (Ma) | (%) |  |
|  |  |  |  |  |  |  |  |  |  |  |  |  |  |  |  |  |  |  |  |  |  |
| **sample MM1 (folded metasandstone)** | | | | | |  |  |  |  |  |  |  |  |  |  |  |  |  |  |  |  |
| a263 |  | 215088 | 82 | 47 | 2.02 | b.d. | 0.38570 | 1.8 | 6.912 | 2.0 | 0.13 | 0.7 | 0.92 | 2103 | 33 | 2100 | 17 | 2098 | 13 | 100 |  |
| a264 |  | 231723 | 91 | 56 | 2.35 | 0.39 | 0.39000 | 1.9 | 7.386 | 2.2 | 0.1373 | 1.1 | 0.87 | 2123 | 34 | 2159 | 20 | 2194 | 19 | 97 |  |
| a265 |  | 1067201 | 460 | 189 | 0.36 | b.d. | 0.37310 | 1.4 | 7.042 | 1.6 | 0.1369 | 0.7 | 0.88 | 2044 | 25 | 2117 | 14 | 2188 | 13 | 93 |  |
| a266 |  | 869466 | 313 | 155 | 0.90 | 0.20 | 0.40440 | 1.1 | 7.968 | 1.2 | 0.1429 | 0.4 | 0.93 | 2189 | 21 | 2227 | 11 | 2263 | 8 | 97 |  |
| a267 |  | 1074440 | 456 | 191 | 0.49 | b.d. | 0.37490 | 1.4 | 6.712 | 1.5 | 0.1298 | 0.5 | 0.95 | 2053 | 25 | 2074 | 13 | 2096 | 9 | 98 |  |
| a268c |  | 1075841 | 487 | 206 | 0.42 | b.d. | 0.38000 | 1.7 | 6.739 | 1.8 | 0.1286 | 0.5 | 0.95 | 2076 | 30 | 2078 | 16 | 2079 | 10 | 100 |  |
| a270 |  | 755863 | 819 | 406 | 1.30 | 0.50 | 0.38450 | 4.8 | 7.047 | 4.9 | 0.1329 | 0.8 | 0.99 | 2097 | 87 | 2117 | 45 | 2137 | 15 | 98 |  |
| a271 |  | 538067 | 508 | 247 | 0.69 | 0.22 | 0.40180 | 1.3 | 7.566 | 1.4 | 0.1366 | 0.6 | 0.90 | 2177 | 24 | 2181 | 13 | 2184 | 11 | 100 |  |
| a272 |  | 461744 | 529 | 232 | 0.36 | 0.39 | 0.39600 | 1.7 | 7.327 | 1.9 | 0.1342 | 0.6 | 0.94 | 2151 | 32 | 2152 | 17 | 2154 | 11 | 100 |  |
| a273 |  | 729451 | 836 | 286 | 0.30 | 0.28 | 0.31440 | 3.0 | 5.432 | 3.1 | 0.1253 | 0.9 | 0.96 | 1762 | 46 | 1890 | 27 | 2033 | 15 | 87 |  |
| a274 |  | 442956 | 528 | 264 | 0.96 | 0.04 | 0.39910 | 1.3 | 7.459 | 1.5 | 0.1355 | 0.6 | 0.92 | 2165 | 25 | 2168 | 13 | 2171 | 10 | 100 |  |
| a275 |  | 89805 | 130 | 47 | 0.67 | 0.80 | 0.31840 | 3.2 | 6.056 | 3.4 | 0.138 | 1.2 | 0.93 | 1782 | 49 | 1984 | 30 | 2202 | 22 | 81 |  |
| a276 |  | 434791 | 529 | 195 | 0.36 | 0.23 | 0.32810 | 1.5 | 6.004 | 1.6 | 0.1327 | 0.6 | 0.92 | 1829 | 24 | 1976 | 14 | 2134 | 11 | 86 |  |
| a277 |  | 368747 | 454 | 191 | 0.80 | b.d. | 0.36690 | 1.6 | 6.595 | 2.0 | 0.1304 | 1.1 | 0.83 | 2015 | 28 | 2059 | 17 | 2103 | 19 | 96 |  |
| a278 |  | 222761 | 181 | 84 | 0.66 | 0.65 | 0.39060 | 3.2 | 7.187 | 3.5 | 0.1334 | 1.2 | 0.94 | 2126 | 59 | 2135 | 31 | 2144 | 21 | 99 |  |
| a279 |  | 267257 | 245 | 119 | 0.71 | b.d. | 0.40840 | 1.3 | 7.768 | 1.4 | 0.1379 | 0.7 | 0.89 | 2208 | 24 | 2205 | 13 | 2202 | 12 | 100 |  |
| a280 |  | 235464 | 263 | 133 | 0.67 | b.d. | 0.42370 | 2.0 | 8.435 | 2.1 | 0.1444 | 0.6 | 0.96 | 2277 | 39 | 2279 | 19 | 2281 | 10 | 100 |  |
| a281 |  | 386352 | 340 | 162 | 0.61 | b.d. | 0.41510 | 1.7 | 8.065 | 1.8 | 0.1409 | 0.6 | 0.94 | 2238 | 32 | 2238 | 16 | 2238 | 11 | 100 |  |
| a282 |  | 576094 | 701 | 254 | 0.44 | b.d. | 0.32470 | 2.7 | 5.673 | 2.7 | 0.1267 | 0.6 | 0.98 | 1813 | 42 | 1927 | 24 | 2053 | 10 | 88 |  |
| a288 |  | 611702 | 551 | 236 | 0.27 | b.d. | 0.39410 | 1.7 | 7.285 | 2.2 | 0.1341 | 1.4 | 0.76 | 2142 | 30 | 2147 | 20 | 2152 | 25 | 100 |  |
| a292 |  | 639648 | 687 | 254 | 0.50 | 0.64 | 0.32340 | 1.6 | 5.672 | 2.7 | 0.1272 | 2.2 | 0.60 | 1806 | 26 | 1927 | 24 | 2060 | 39 | 88 |  |
| a293 |  | 488750 | 513 | 198 | 0.29 | 0.27 | 0.35460 | 3.1 | 6.363 | 3.2 | 0.1301 | 0.8 | 0.97 | 1957 | 53 | 2027 | 29 | 2100 | 13 | 93 |  |
| a294 |  | 145004 | 132 | 62 | 0.66 | 1.05 | 0.39850 | 2.6 | 7.542 | 2.9 | 0.1373 | 1.1 | 0.92 | 2162 | 49 | 2178 | 26 | 2193 | 19 | 99 |  |
| a295c |  | 219275 | 212 | 103 | 0.77 | 0.08 | 0.40480 | 1.8 | 7.65 | 1.9 | 0.1371 | 0.6 | 0.96 | 2191 | 34 | 2191 | 17 | 2190 | 10 | 100 |  |
| a298 |  | 636730 | 596 | 260 | 0.60 | 0.35 | 0.37870 | 2.4 | 6.737 | 2.5 | 0.129 | 0.8 | 0.95 | 2070 | 42 | 2078 | 22 | 2085 | 13 | 99 |  |
| a299 |  | 642491 | 649 | 352 | 1.22 | 0.32 | 0.40740 | 1.6 | 7.776 | 1.7 | 0.1384 | 0.6 | 0.94 | 2203 | 31 | 2205 | 16 | 2207 | 10 | 100 |  |
| a301 |  | 206469 | 220 | 90 | 0.74 | b.d. | 0.35760 | 2.3 | 6.31 | 2.7 | 0.128 | 1.5 | 0.83 | 1971 | 39 | 2020 | 24 | 2070 | 27 | 95 |  |
| a302 |  | 402210 | 286 | 135 | 0.93 | 1.63 | 0.38850 | 2.1 | 7.184 | 2.7 | 0.1341 | 1.7 | 0.79 | 2116 | 39 | 2135 | 25 | 2152 | 29 | 98 |  |
| a305 |  | 616377 | 531 | 243 | 0.76 | b.d. | 0.37940 | 1.5 | 6.715 | 2.2 | 0.1284 | 1.7 | 0.66 | 2073 | 26 | 2075 | 20 | 2076 | 30 | 100 |  |
| a306 |  | 484717 | 454 | 214 | 0.59 | b.d. | 0.40390 | 1.8 | 7.604 | 1.9 | 0.1365 | 0.5 | 0.96 | 2187 | 33 | 2185 | 17 | 2184 | 10 | 100 |  |
| a307 |  | 413737 | 395 | 165 | 0.31 | 0.64 | 0.37830 | 3.9 | 6.694 | 4.0 | 0.1283 | 1 | 0.97 | 2068 | 70 | 2072 | 36 | 2075 | 17 | 100 |  |
| a308 |  | 246762 | 207 | 117 | 1.23 | b.d. | 0.42290 | 2.3 | 8.353 | 2.4 | 0.1433 | 0.5 | 0.97 | 2274 | 45 | 2270 | 22 | 2267 | 9 | 100 |  |
| a309 |  | 378268 | 488 | 160 | 0.11 | b.d. | 0.31120 | 1.6 | 5.491 | 1.9 | 0.1279 | 1 | 0.85 | 1747 | 25 | 1899 | 17 | 2070 | 18 | 84 |  |
| a310 |  | 582647 | 590 | 261 | 0.65 | 0.44 | 0.38040 | 1.2 | 6.729 | 1.4 | 0.1283 | 0.7 | 0.88 | 2078 | 22 | 2076 | 13 | 2075 | 12 | 100 |  |
| a311 |  | 150593 | 150 | 68 | 0.45 | b.d. | 0.40570 | 1.3 | 7.733 | 1.5 | 0.1383 | 0.8 | 0.85 | 2195 | 24 | 2200 | 14 | 2206 | 14 | 100 |  |
| a312 |  | 932540 | 840 | 384 | 0.65 | b.d. | 0.38840 | 2.1 | 7.023 | 2.2 | 0.1312 | 0.7 | 0.95 | 2115 | 37 | 2114 | 20 | 2114 | 12 | 100 |  |
| a313 |  | 236336 | 209 | 100 | 0.66 | 0.15 | 0.40650 | 1.5 | 7.757 | 1.7 | 0.1384 | 0.8 | 0.89 | 2199 | 29 | 2203 | 16 | 2207 | 14 | 100 |  |
| a315 |  | 342762 | 435 | 151 | 0.33 | b.d. | 0.31330 | 6.0 | 5.387 | 6.1 | 0.1247 | 1.1 | 0.98 | 1757 | 93 | 1883 | 54 | 2025 | 19 | 87 |  |
| a316 |  | 588475 | 500 | 230 | 0.50 | b.d. | 0.40610 | 1.1 | 7.676 | 1.2 | 0.1371 | 0.5 | 0.90 | 2197 | 20 | 2194 | 11 | 2191 | 9 | 100 |  |
| a319 |  | 195145 | 167 | 69 | 0.73 | 1.47 | 0.34650 | 2.1 | 6.504 | 2.4 | 0.1361 | 1.1 | 0.88 | 1918 | 35 | 2046 | 21 | 2178 | 20 | 88 |  |
| a322 |  | 191234 | 196 | 89 | 0.47 | b.d. | 0.40250 | 0.9 | 7.581 | 1.2 | 0.1366 | 0.8 | 0.75 | 2181 | 16 | 2183 | 11 | 2185 | 14 | 100 |  |
| a323 |  | 217836 | 188 | 96 | 0.90 | 0.08 | 0.40690 | 4.2 | 7.875 | 4.3 | 0.1404 | 1.1 | 0.97 | 2201 | 79 | 2217 | 40 | 2232 | 19 | 99 |  |
| a324 |  | 443861 | 479 | 213 | 0.32 | b.d. | 0.40380 | 1.2 | 7.603 | 1.3 | 0.1366 | 0.5 | 0.91 | 2186 | 21 | 2185 | 11 | 2184 | 9 | 100 |  |
| a325 |  | 480182 | 401 | 182 | 0.50 | b.d. | 0.39350 | 2.7 | 7.268 | 2.8 | 0.134 | 0.9 | 0.95 | 2139 | 49 | 2145 | 26 | 2151 | 16 | 99 |  |
| a326 |  | 186706 | 194 | 93 | 0.53 | b.d. | 0.41540 | 1.0 | 7.924 | 1.2 | 0.1384 | 0.7 | 0.79 | 2239 | 18 | 2222 | 11 | 2207 | 13 | 101 |  |
| a327 |  | 322567 | 380 | 147 | 0.88 | 0.24 | 0.31910 | 3.3 | 5.685 | 3.4 | 0.1292 | 0.7 | 0.98 | 1785 | 52 | 1929 | 30 | 2087 | 12 | 86 |  |
| a334c |  | 489648 | 414 | 190 | 0.45 | 1.12 | 0.40770 | 2.1 | 7.736 | 2.3 | 0.1376 | 1 | 0.91 | 2205 | 39 | 2201 | 21 | 2197 | 17 | 100 |  |
| **sample MM-6 (granodiorite)** | | | | |  |  |  |  |  |  |  |  |  |  |  |  |  |  |  |  |  |
| a166 |  | 1690697 | 1269 | 332 | 0.06 | 1.10 | 0.25350 | 1.1 | 3.99 | 1.9 | 0.1142 | 1.5 | 0.59 | 1456 | 14 | 1632 | 15 | 1867 | 27 | 78 |  |
| a167 |  | 793052 | 964 | 333 | 0.02 | 1.52 | 0.33510 | 3.9 | 5.26 | 4.5 | 0.1138 | 2.3 | 0.86 | 1863 | 63 | 1862 | 39 | 1861 | 41 | 100 |  |
| a168 |  | 964114 | 1114 | 379 | 0.04 | b.d. | 0.33660 | 1.5 | 5.304 | 1.7 | 0.1143 | 0.6 | 0.93 | 1870 | 25 | 1869 | 14 | 1869 | 11 | 100 |  |
| a169 |  | 2334705 | 2891 | 986 | 0.08 | b.d. | 0.33490 | 3.3 | 5.274 | 3.4 | 0.1142 | 0.8 | 0.97 | 1862 | 54 | 1865 | 29 | 1868 | 15 | 100 |  |
| a174 |  | 1193121 | 1990 | 609 | 0.10 | 0.81 | 0.29470 | 1.6 | 4.629 | 1.9 | 0.1139 | 1 | 0.85 | 1665 | 24 | 1755 | 16 | 1863 | 18 | 89 |  |
| a179 |  | 566430 | 1100 | 387 | 0.27 | 1.43 | 0.32950 | 1.1 | 5.135 | 3.7 | 0.113 | 3.5 | 0.31 | 1836 | 18 | 1842 | 32 | 1849 | 64 | 99 |  |
| a182 |  | 583034 | 827 | 284 | 0.08 | 0.81 | 0.33320 | 3.9 | 5.209 | 4.1 | 0.1134 | 1.2 | 0.95 | 1854 | 64 | 1854 | 36 | 1854 | 22 | 100 |  |
| a183 |  | 1364293 | 1417 | 482 | 0.05 | 0.54 | 0.32400 | 4.2 | 5.099 | 4.7 | 0.1141 | 2.2 | 0.88 | 1809 | 66 | 1836 | 41 | 1866 | 40 | 97 |  |
| **Reference materials** | | | | | |  |  |  |  |  |  |  |  |  |  |  |  |  |  |  |  |
| **GJ1** | | |  |  |  |  |  |  |  |  |  |  |  |  |  |  |  |  |  |  |  |
| GJ1-1 |  | 178171 | 286 | 27 | 0.03 | 0.05 | 0.09776 | 1.0 | 0.8114 | 1.5 | 0.0602 | 1.1 | 0.67 | 601 | 6 | 603 | 7 | 611 | 24 | 98 |  |
| GJ1-2 |  | 176665 | 283 | 26 | 0.03 | 0.05 | 0.09792 | 1.0 | 0.8127 | 1.5 | 0.06019 | 1 | 0.70 | 602 | 6 | 604 | 7 | 611 | 22 | 99 |  |
| GJ1-3 |  | 173325 | 279 | 26 | 0.03 | 0.03 | 0.09864 | 0.9 | 0.8126 | 1.3 | 0.05975 | 0.9 | 0.72 | 606 | 5 | 604 | 6 | 594 | 20 | 102 |  |
| GJ1-4 |  | 172837 | 276 | 26 | 0.03 | 0.04 | 0.09830 | 1.0 | 0.814 | 1.6 | 0.06006 | 1.3 | 0.60 | 604 | 6 | 605 | 7 | 606 | 28 | 100 |  |
| GJ1-5 |  | 172861 | 279 | 26 | 0.03 | 0.04 | 0.09867 | 0.9 | 0.821 | 1.5 | 0.06035 | 1.2 | 0.63 | 607 | 5 | 609 | 7 | 616 | 25 | 98 |  |
| GJ1-6 |  | 173781 | 279 | 26 | 0.03 | 0.03 | 0.09773 | 1.0 | 0.8082 | 1.4 | 0.05998 | 1 | 0.69 | 601 | 6 | 601 | 6 | 603 | 22 | 100 |  |
| GJ1-7 |  | 170674 | 274 | 26 | 0.03 | 0.06 | 0.09789 | 1.0 | 0.8112 | 1.3 | 0.06011 | 0.9 | 0.73 | 602 | 6 | 603 | 6 | 607 | 20 | 99 |  |
| GJ1-8 |  | 170220 | 275 | 26 | 0.03 | 0.05 | 0.09762 | 0.9 | 0.8054 | 1.3 | 0.05984 | 1 | 0.70 | 600 | 5 | 600 | 6 | 598 | 21 | 100 |  |
| GJ1-9 |  | 171157 | 279 | 26 | 0.03 | 0.07 | 0.09905 | 1.0 | 0.8211 | 1.5 | 0.06012 | 1.1 | 0.67 | 609 | 6 | 609 | 7 | 608 | 23 | 100 |  |
| GJ1-10 |  | 168663 | 279 | 26 | 0.03 | 0.03 | 0.09757 | 1.0 | 0.8066 | 1.7 | 0.05996 | 1.3 | 0.58 | 600 | 6 | 601 | 8 | 602 | 29 | 100 |  |
| GJ1-11 |  | 170475 | 284 | 27 | 0.03 | 0.06 | 0.09879 | 1.0 | 0.8094 | 1.4 | 0.05943 | 1 | 0.71 | 607 | 6 | 602 | 6 | 583 | 21 | 104 |  |
| GJ1-12 |  | 173661 | 300 | 28 | 0.03 | 0.03 | 0.09860 | 1.0 | 0.8115 | 1.4 | 0.05969 | 1 | 0.71 | 606 | 6 | 603 | 7 | 592 | 22 | 102 |  |
| GJ1-13 |  | 172637 | 280 | 26 | 0.03 | 0.05 | 0.09849 | 0.9 | 0.8177 | 1.4 | 0.06021 | 1 | 0.67 | 606 | 5 | 607 | 6 | 611 | 22 | 99 |  |
| GJ1-14 |  | 171403 | 280 | 26 | 0.03 | 0.06 | 0.09820 | 0.9 | 0.8132 | 1.5 | 0.06006 | 1.1 | 0.63 | 604 | 5 | 604 | 7 | 606 | 25 | 100 |  |
| GJ1-15 |  | 168655 | 275 | 26 | 0.03 | 0.05 | 0.09855 | 1.0 | 0.8131 | 1.3 | 0.05984 | 0.8 | 0.77 | 606 | 6 | 604 | 6 | 598 | 18 | 101 |  |
| GJ1-16 |  | 171844 | 278 | 26 | 0.03 | 0.04 | 0.09795 | 0.9 | 0.8121 | 1.3 | 0.06013 | 1 | 0.71 | 602 | 5 | 604 | 6 | 608 | 21 | 99 |  |
| GJ1-17 |  | 169693 | 279 | 26 | 0.03 | 0.05 | 0.09789 | 1.0 | 0.812 | 1.6 | 0.06016 | 1.3 | 0.61 | 602 | 6 | 604 | 7 | 609 | 27 | 99 |  |
| GJ1-18 |  | 169361 | 278 | 26 | 0.03 | 0.06 | 0.09834 | 1.0 | 0.7976 | 1.6 | 0.05882 | 1.2 | 0.65 | 605 | 6 | 595 | 7 | 561 | 26 | 108 |  |
| GJ1-19 |  | 167742 | 275 | 26 | 0.03 | 0.02 | 0.09787 | 1.0 | 0.8167 | 1.4 | 0.06052 | 1 | 0.72 | 602 | 6 | 606 | 6 | 622 | 21 | 97 |  |
| GJ1-20 |  | 166713 | 272 | 26 | 0.03 | 0.06 | 0.09874 | 0.9 | 0.8125 | 1.3 | 0.05968 | 0.9 | 0.71 | 607 | 5 | 604 | 6 | 592 | 21 | 103 |  |
| GJ1-21 |  | 170877 | 282 | 26 | 0.03 | 0.03 | 0.09747 | 0.9 | 0.8101 | 1.3 | 0.06028 | 0.9 | 0.71 | 600 | 5 | 603 | 6 | 614 | 20 | 98 |  |
| GJ1-22 |  | 171321 | 282 | 26 | 0.03 | 0.04 | 0.09839 | 0.9 | 0.8151 | 1.4 | 0.06008 | 1 | 0.68 | 605 | 5 | 605 | 6 | 606 | 21 | 100 |  |
| GJ1-23 |  | 171625 | 289 | 27 | 0.03 | 0.05 | 0.09851 | 0.9 | 0.8152 | 1.3 | 0.06002 | 0.9 | 0.71 | 606 | 5 | 605 | 6 | 604 | 19 | 100 |  |
| GJ1-24 |  | 169886 | 276 | 26 | 0.03 | 0.02 | 0.09818 | 0.9 | 0.8112 | 1.4 | 0.05993 | 1.1 | 0.65 | 604 | 5 | 603 | 6 | 601 | 23 | 100 |  |
| GJ1-25 |  | 169245 | 281 | 26 | 0.03 | 0.04 | 0.09823 | 1.0 | 0.8137 | 1.5 | 0.06008 | 1.1 | 0.67 | 604 | 6 | 605 | 7 | 606 | 24 | 100 |  |
| GJ1-26 |  | 170371 | 281 | 26 | 0.03 | 0.05 | 0.09785 | 1.0 | 0.8093 | 1.4 | 0.05999 | 1 | 0.71 | 602 | 6 | 602 | 6 | 603 | 21 | 100 |  |
| mean (n=26): | | |  |  |  |  | 0.0982 |  | 0.8121 |  | 0.0600 |  |  | 604 |  | 604 |  | 603 |  |  |  |
| 2SD(abs): | | |  |  |  |  | 0.0009 |  | 0.0095 |  | 0.0007 |  |  | 5 |  | 5 |  | 24 |  |  |  |
| 2SD(%) |  |  |  |  |  |  | 0.87 |  | 1.17 |  | 1.10 |  |  |  |  |  |  |  |  |  |  |
| **Plešovice** | | |  |  |  |  |  |  |  |  |  |  |  |  |  |  |  |  |  |  |  |
| a03 |  | 191739 | 559 | 29 | 0.13 | 0.13 | 0.05394 | 1.0 | 0.3905 | 1.7 | 0.05251 | 1.4 | 0.55 | 339 | 3 | 335 | 5 | 308 | 33 | 110 |  |
| a04 |  | 196268 | 562 | 29 | 0.13 | 0.21 | 0.05348 | 1.0 | 0.3932 | 1.4 | 0.05332 | 1.1 | 0.66 | 336 | 3 | 337 | 4 | 342 | 25 | 98 |  |
| a35 |  | 197851 | 573 | 30 | 0.14 | b.d. | 0.05377 | 1.0 | 0.3945 | 1.6 | 0.05321 | 1.2 | 0.61 | 338 | 3 | 338 | 5 | 338 | 28 | 100 |  |
| a36 |  | 204469 | 587 | 31 | 0.14 | 0.00 | 0.05414 | 0.9 | 0.3964 | 1.2 | 0.05311 | 0.8 | 0.76 | 340 | 3 | 339 | 4 | 333 | 18 | 102 |  |
| a69 |  | 207372 | 580 | 31 | 0.15 | 0.25 | 0.05381 | 1.0 | 0.3956 | 1.7 | 0.05332 | 1.4 | 0.58 | 338 | 3 | 338 | 5 | 342 | 31 | 99 |  |
| a70 |  | 279702 | 765 | 40 | 0.13 | 0.12 | 0.05357 | 1.0 | 0.3949 | 1.4 | 0.05346 | 1 | 0.70 | 336 | 3 | 338 | 4 | 348 | 23 | 97 |  |
| a104 |  | 271805 | 694 | 37 | 0.13 | 0.65 | 0.05468 | 1.2 | 0.4009 | 1.8 | 0.05317 | 1.4 | 0.67 | 343 | 4 | 342 | 5 | 336 | 31 | 102 |  |
| a105 |  | 243056 | 675 | 35 | 0.12 | 0.85 | 0.05362 | 1.0 | 0.3928 | 2.0 | 0.05313 | 1.7 | 0.52 | 337 | 3 | 336 | 6 | 334 | 39 | 101 |  |
| a148 |  | 197799 | 579 | 30 | 0.11 | b.d. | 0.05400 | 1.0 | 0.3968 | 1.5 | 0.0533 | 1.1 | 0.65 | 339 | 3 | 339 | 4 | 342 | 25 | 99 |  |
| a149 |  | 192529 | 553 | 29 | 0.11 | 0.00 | 0.05432 | 1.0 | 0.4002 | 1.4 | 0.05343 | 1 | 0.69 | 341 | 3 | 342 | 4 | 347 | 23 | 98 |  |
| a196 |  | 106512 | 351 | 19 | 0.09 | 0.19 | 0.05464 | 0.9 | 0.4007 | 1.7 | 0.05319 | 1.5 | 0.53 | 343 | 3 | 342 | 5 | 337 | 33 | 102 |  |
| a197 |  | 125958 | 383 | 20 | 0.10 | 0.26 | 0.05398 | 1.1 | 0.3967 | 1.9 | 0.0533 | 1.5 | 0.59 | 339 | 4 | 339 | 5 | 342 | 34 | 99 |  |
| a240 |  | 185381 | 538 | 28 | 0.12 | b.d. | 0.05398 | 1.7 | 0.3972 | 2.1 | 0.05337 | 1.2 | 0.83 | 339 | 6 | 340 | 6 | 344 | 26 | 98 |  |
| a241 |  | 283850 | 965 | 51 | 0.09 | 0.41 | 0.05400 | 2.2 | 0.3993 | 2.5 | 0.05363 | 1.2 | 0.87 | 339 | 7 | 341 | 7 | 355 | 28 | 95 |  |
| a285 |  | 225890 | 668 | 35 | 0.11 | 0.15 | 0.05401 | 1.0 | 0.3996 | 1.6 | 0.05366 | 1.2 | 0.62 | 339 | 3 | 341 | 5 | 357 | 28 | 95 |  |
| a286 |  | 227347 | 648 | 34 | 0.12 | 0.19 | 0.05373 | 1.0 | 0.3938 | 1.3 | 0.05317 | 0.9 | 0.72 | 337 | 3 | 337 | 4 | 336 | 21 | 100 |  |
| a330 |  | 273673 | 813 | 42 | 0.12 | 0.16 | 0.05359 | 1.0 | 0.393 | 1.5 | 0.05319 | 1.1 | 0.67 | 337 | 3 | 337 | 4 | 337 | 26 | 100 |  |
| a331 |  | 269357 | 789 | 41 | 0.12 | 0.10 | 0.05413 | 1.0 | 0.3973 | 1.4 | 0.05324 | 0.9 | 0.73 | 340 | 3 | 340 | 4 | 339 | 21 | 100 |  |
| a385 |  | 238102 | 706 | 37 | 0.11 | 0.00 | 0.05421 | 1.0 | 0.3973 | 1.4 | 0.05315 | 1 | 0.70 | 340 | 3 | 340 | 4 | 335 | 22 | 102 |  |
| a386 |  | 226135 | 666 | 35 | 0.11 | 0.00 | 0.05391 | 1.0 | 0.3958 | 1.6 | 0.05325 | 1.3 | 0.61 | 338 | 3 | 339 | 5 | 340 | 28 | 100 |  |
| a441 |  | 180256 | 551 | 29 | 0.12 | 0.00 | 0.05382 | 0.9 | 0.3942 | 1.4 | 0.05312 | 1 | 0.70 | 338 | 3 | 337 | 4 | 334 | 22 | 101 |  |
| a442 |  | 183777 | 558 | 29 | 0.12 | 0.00 | 0.05375 | 0.9 | 0.3941 | 1.4 | 0.05317 | 1.1 | 0.65 | 338 | 3 | 337 | 4 | 336 | 25 | 100 |  |
| a496 |  | 179584 | 533 | 28 | 0.13 | 0.19 | 0.05403 | 1.0 | 0.3969 | 1.6 | 0.05328 | 1.3 | 0.60 | 339 | 3 | 339 | 5 | 341 | 30 | 100 |  |
| a497 |  | 182671 | 538 | 28 | 0.13 | 0.19 | 0.05384 | 1.0 | 0.3968 | 1.4 | 0.05346 | 1.1 | 0.67 | 338 | 3 | 339 | 4 | 348 | 24 | 97 |  |
| a551 |  | 183653 | 510 | 27 | 0.14 | b.d. | 0.05389 | 1.0 | 0.394 | 1.4 | 0.05302 | 1 | 0.70 | 338 | 3 | 337 | 4 | 330 | 23 | 103 |  |
| a552 |  | 182419 | 544 | 28 | 0.11 | 0.00 | 0.05395 | 1.1 | 0.3968 | 1.6 | 0.05334 | 1.2 | 0.68 | 339 | 4 | 339 | 5 | 343 | 26 | 99 |  |
| mean (n=26): | | |  |  |  |  | 0.0540 |  | 0.3961 |  | 0.0533 |  |  | 339 |  | 339 |  | 339 |  |  |  |
| 2SD(abs): | | |  |  |  |  | 0.0006 |  | 0.0052 |  | 0.0004 |  |  | 4 |  | 4 |  | 18 |  |  |  |
| 2SD(%) |  |  |  |  |  |  | 1.07 |  | 1.32 |  | 0.81 |  |  |  |  |  |  |  |  |  |  |
| **OG1** | | |  |  |  |  |  |  |  |  |  |  |  |  |  |  |  |  |  |  |  |
| a05 |  | 477578 | 102 | 99 | 0.90 | 0.95 | 0.71260 | 1.0 | 29.39 | 1.1 | 0.2991 | 0.5 | 0.89 | 3468 | 27 | 3467 | 11 | 3466 | 8 | 100 |  |
| a37 |  | 527676 | 107 | 104 | 0.97 | 1.25 | 0.71150 | 1.0 | 29.37 | 1.2 | 0.2993 | 0.5 | 0.88 | 3464 | 28 | 3466 | 11 | 3467 | 8 | 100 |  |
| a71 |  | 558814 | 123 | 124 | 1.26 | 1.15 | 0.71260 | 0.9 | 29.23 | 1.1 | 0.2975 | 0.6 | 0.85 | 3468 | 25 | 3461 | 11 | 3457 | 9 | 100 |  |
| a106 |  | 592799 | 142 | 135 | 0.84 | 0.21 | 0.70670 | 1.6 | 29.06 | 1.7 | 0.2982 | 0.5 | 0.95 | 3446 | 42 | 3455 | 16 | 3461 | 8 | 100 |  |
| a150 |  | 493903 | 84 | 84 | 1.15 | b.d. | 0.70790 | 1.4 | 29.06 | 1.4 | 0.2978 | 0.3 | 0.97 | 3451 | 36 | 3456 | 14 | 3459 | 5 | 100 |  |
| a198 |  | 603943 | 154 | 145 | 0.70 | 0.38 | 0.71980 | 0.9 | 29.59 | 0.9 | 0.2982 | 0.4 | 0.93 | 3495 | 24 | 3473 | 9 | 3461 | 5 | 101 |  |
| a287 |  | 494580 | 95 | 97 | 1.32 | b.d. | 0.70960 | 1.1 | 29.1 | 1.2 | 0.2974 | 0.4 | 0.93 | 3457 | 30 | 3457 | 12 | 3457 | 7 | 100 |  |
| a332 |  | 1071948 | 258 | 265 | 1.23 | 1.88 | 0.71220 | 1.1 | 29.4 | 1.3 | 0.2994 | 0.7 | 0.85 | 3467 | 29 | 3467 | 12 | 3467 | 10 | 100 |  |
| a387 |  | 613228 | 141 | 140 | 1.16 | 1.13 | 0.71010 | 1.0 | 29.15 | 1.1 | 0.2977 | 0.5 | 0.90 | 3459 | 26 | 3459 | 11 | 3458 | 8 | 100 |  |
| a443 |  | 477226 | 112 | 112 | 1.12 | 0.27 | 0.70950 | 1.3 | 29.22 | 1.4 | 0.2987 | 0.4 | 0.97 | 3456 | 35 | 3461 | 13 | 3464 | 6 | 100 |  |
| a553 |  | 1168935 | 254 | 258 | 1.25 | 1.34 | 0.70890 | 1.0 | 29.13 | 1.1 | 0.298 | 0.5 | 0.89 | 3454 | 26 | 3458 | 11 | 3460 | 8 | 100 |  |
| a242* |  | 601726 | 205 | 160 | 0.34 | 1.54 | 0.64650 | 1.7 | 22.41 | 2.2 | 0.2514 | 1.5 | 0.74 | 3215 | 42 | 3202 | 22 | 3194 | 24 | 101 |  |
| a498* |  | 861268 | 183 | 186 | 1.33 | 3.22 | 0.69490 | 1.8 | 28.17 | 2.0 | 0.294 | 1 | 0.87 | 3401 | 46 | 3425 | 20 | 3439 | 16 | 99 |  |
| mean (n=11 of 13): | | |  |  |  |  | 0.7110 |  | 29.2455 |  | 0.2983 |  |  | 3462 |  | 3462 |  | 3461 |  |  |  |
| 2SD(abs): | | |  |  |  |  | 0.0070 |  | 0.3419 |  | 0.0014 |  |  | 26 |  | 12 |  | 7 |  |  |  |
| 2SD(%) |  |  |  |  |  |  | 0.98 |  | 1.17 |  | 0.48 |  |  |  |  |  |  |  |  |  |  |
| *Standard not used. | | |  |  |  |  |  |  |  |  |  |  |  |  |  |  |  |  |  |  |  |
| Spot size = 30µm; depth of crater = ~20µm. ^206^Pb/^238^U error = quadratic additions of within-run precision (2 SE) + external reproducibility (2 SD) of the reference zircon. For ^207^Pb/^206^Pb error propagation (^207^Pb signal dependent), see Ref. 4. ^207^Pb/^235^U error = quadratic addition of ^207^Pb/^206^Pb and ^206^Pb/^238^U uncertainties. | | | | | | | | | | | | | | | | | | | | | |
|  |  |  |  |  |  |  |  |  |  |  |  |  |  |  |  |  |  |  |  |  |  |
|  |  |  |  |  |  |  |  |  |  |  |  |  |  |  |  |  |  |  |  |  |  |
| ^a^ Within-run background-corrected mean ^207^Pb signal in cps (counts per second). | | | | | | | | | | |  |  |  |  |  |  |  |  |  |  |  |
| ^b^ Uranium and Pb content and Th/U ratio were calculated relative to GJ-1 reference zircon. | | | | | | | | | | | | | | | | | | | | | |
| ^c^ Percentage of common Pb in ^206^Pb. b.d. = below dectection limit. | | | | | | | | | |  |  |  |  |  |  |  |  |  |  |  |  |
| ^d^ Corrected for background, within-run Pb–U fractionation (^206^Pb/^238^U) and common Pb using model Pb composition^5^, | | | | | | | | | | | | | | | | | | | | |  |
| subsequently normalised to GJ-1 (ID-TIMS value/measured value); ^207^Pb/^235^U calculated using ^207^Pb/^206^Pb/(^238^U/^206^Pb*1/137.88). | | | | | | | | | | | | | | | | |  |  |  |  |  |
| ^e^ Rho = ^206^Pb/^238^U/^207^Pb/^235^U error correlation coefficient. | | | | | | | |  |  |  |  |  |  |  |  |  |  |  |  |  |  |
| ^f^  Degree of concordance = ^206^Pb/^238^U age / ^207^Pb/^206^Pb age x 100. | | | | | | | | |  |  |  |  |  |  |  |  |  |  |  |  |  |

Supplementary Table S2. Results of whole-rock chemical analyses of queluzite and graphitic schist

|  |  | Queluzite | |  |  |  |  |  |  |  |  | Mn-rich graphitic schist | | |  | Graphitic schist | | | | |
| --- | --- | --- | --- | --- | --- | --- | --- | --- | --- | --- | --- | --- | --- | --- | --- | --- | --- | --- | --- | --- |
|  |  | **MM-9** | **MM-10** | **MM-11** | **MM-12** | **MM-14** | **MM-15** | **MM-16** | **MM-25** | **MM-26** | **MM-27** | **MM-22** | **MM-23c** | **MM-24a** | **MM-24b** | **MM-20** | **MM-21** | **MM-23a** | **MM-23b** | **MM-28** |
| δ^13^C-GRA | ‰ | -14.8 | -14.5 | -18.8 | -17.5 | -16.9 | -20.4 | -20.8 |  |  |  |  |  |  |  | -22.5 | -20.8 |  |  |  |
| δ^13^C-CAR | ‰ | -9.0 | -10.0 | -14.1 | -10.8 | -11.9 | -10.2 |  |  |  |  |  |  |  |  |  |  |  |  |  |
| δ^98^Mo | ‰ | -1.03 | -0.86 | -1.80 | -0.92 | -0.59 | -0.69 | -0.47 | -1.55 | -1.29 | -0.74 | -0.93 | -0.44 |  | -1.14 | 0.80 | 0.05 | 0.07 | 0.31 | -0.17 |
| SiO_2_ | % | 16.91 | 15.82 | 27.97 | 23.59 | 35.61 | 38.12 | 44.04 | 11.48 | 19.55 | 30.01 | 54.04 | 48.61 | 51.39 | 50.97 | 49.46 | 53.27 | 54.23 | 52.59 | 54.68 |
| Al_2_O_3_ | % | 7.04 | 5.41 | 8.04 | 5.95 | 5.31 | 9.23 | 12.72 | 4.57 | 4.88 | 7.29 | 8.99 | 12.26 | 12.99 | 11.31 | 10.91 | 12.14 | 11.46 | 12.4 | 14.79 |
| Al | % | 3.73 | 2.86 | 4.26 | 3.15 | 2.81 | 4.89 | 6.73 | 2.42 | 2.58 | 3.86 | 4.76 | 6.49 | 6.88 | 5.99 | 5.78 | 6.43 | 6.07 | 6.56 | 7.83 |
| Fe_2_O_3_ | % | 3.92 | 3.57 | 5.37 | 4.53 | 3.31 | 5.82 | 7.27 | 2.47 | 3.51 | 3.97 | 4.13 | 6.56 | 6.91 | 7.28 | 8.93 | 6.19 | 6.63 | 6.1 | 9.19 |
| Fe | % | 2.74 | 2.50 | 3.76 | 3.17 | 2.31 | 4.07 | 5.08 | 1.73 | 2.45 | 2.78 | 2.89 | 4.59 | 4.83 | 5.09 | 6.24 | 4.33 | 4.64 | 4.27 | 6.43 |
| MgO | % | 3.18 | 4.19 | 3.91 | 4.08 | 5.25 | 3.04 | 2.22 | 4.53 | 4.37 | 3.15 | 2.54 | 3.26 | 4.44 | 5.17 | 3.14 | 1.82 | 3.29 | 3.32 | 5.21 |
| CaO | % | 6.33 | 5.93 | 7.05 | 7.39 | 5.00 | 6.33 | 4.44 | 4.98 | 4.99 | 5.57 | 2.99 | 2.76 | 3.12 | 4.27 | 2.36 | 0.74 | 1.94 | 2.36 | 3.4 |
| Na_2_O | % | <0.01 | <0.01 | <0.01 | <0.01 | <0.01 | <0.01 | <0.01 | <0.01 | <0.01 | 0.03 | 0.02 | 1.51 | 2.48 | 1.62 | 3.70 | 3.64 | 3.22 | 4.09 | 1.91 |
| K_2_O | % | <0.01 | <0.01 | <0.01 | <0.01 | <0.01 | <0.01 | <0.01 | 0.21 | 0.28 | 0.03 | 0.02 | 3.79 | 2.61 | 1.92 | 1.55 | 4.04 | 3.09 | 2.38 | 1.87 |
| TiO_2_ | % | 0.33 | 0.24 | 0.41 | 0.31 | 0.23 | 0.47 | 0.65 | 0.20 | 0.23 | 0.33 | 0.81 | 0.64 | 0.8 | 0.67 | 0.89 | 0.53 | 0.63 | 0.73 | 1.06 |
| P_2_O_5_ | % | 0.13 | 0.13 | 0.13 | 0.13 | 0.19 | 0.10 | 0.16 | 0.13 | <0.01 | 0.13 | 0.32 | 0.25 | 0.41 | 0.43 | 0.25 | 0.09 | 0.23 | 0.31 | 0.24 |
| MnO | % | 42.38 | 42.57 | 35.45 | 42.46 | 39.26 | 26.90 | 18.81 | 34.36 | 36.39 | 32.86 | 15.48 | 5.19 | 2.67 | 4.62 | 0.15 | 0.92 | 0.93 | 0.67 | 0.13 |
| Mn | % | 32.83 | 32.97 | 27.46 | 32.89 | 30.41 | 20.84 | 14.57 | 26.62 | 28.19 | 25.45 | 11.99 | 4.02 | 2.07 | 3.58 | 0.12 | 0.71 | 0.72 | 0.52 | 0.10 |
| Fe/Mn |  | 0.08 | 0.08 | 0.14 | 0.10 | 0.08 | 0.20 | 0.35 | 0.06 | 0.09 | 0.11 | 0.24 | 1.1 | 2.3 | 1.4 | 53.7 | 6.1 | 6.4 | 8.2 | 63.8 |
| TOT-C | % | 9.54 | 10.33 | 8.68 | 8.75 | 3.48 | 8.96 | 7.16 | 10.73 | 8.10 | 5.27 | 10.14 | 13.74 | 10.06 | 9.59 | 16.79 | 14.74 | 11.18 | 12.90 | 3.90 |
| C-GRA | % | 3.31 | 3.60 | 5.54 | 4.96 | 1.53 | 7.95 | 6.39 | 2.70 | 3.15 | 3.08 | 8.18 | 11.96 | 8.79 | 8.18 | 15.59 | 14.09 | 9.76 | 10.97 | 3.36 |
| TOT-S | % | 0.78 | 0.33 | 0.28 | 1.4 | 0.25 | 1.58 | 3.05 | 0.38 | 0.39 | 0.19 | 0.7 | 1.78 | 2.22 | 2.16 | 3.47 | 2.5 | 2.93 | 2.35 | 3.34 |
| Cr_2_O_3_ | % | 0.009 | 0.005 | 0.014 | 0.008 | 0.005 | 0.02 | 0.023 | 0.012 | 0.013 | 0.018 | 0.044 | 0.036 | 0.037 | 0.035 | 0.025 | 0.024 | 0.029 | 0.032 | 0.04 |
| LOI | % | 19.4 | 21.8 | 11.3 | 11.3 | 5.5 | 9.8 | 9.4 | 24.8 | 15.1 | 7.4 | 10.4 | 14.3 | 11.6 | 11.2 | 18.3 | 15.8 | 13.8 | 14.6 | 7 |
| Be | ppm | <1 | <1 | <1 | <1 | <1 | <1 | <1 | <1 | 3 | 3 | <1 | 1 | <1 | <1 | 2 | 2 | <1 | 1 | <1 |
| Sc | ppm | 9 | 7 | 12 | 9 | 7 | 14 | 22 | 6 | 7 | 10 | 18 | 18 | 21 | 19 | 21 | 16 | 16 | 17 | 27 |
| V | ppm | 350 | 307 | 301 | 345 | 381 | 192 | 259 | 441 | 366 | 399 | 175 | 199 | 202 | 233 | 223 | 153 | 168 | 139 | 164 |
| Co | ppm | 308 | 313 | 329 | 267 | 416 | 132 | 273 | 406 | 348 | 323 | 135.4 | 85.6 | 118.1 | 120.7 | 95 | 118 | 75.3 | 73.1 | 39.1 |
| Ni | ppm | 1188 | 1009 | 1196 | 900 | 751 | 260 | 535 | 877 | 1055 | 1219 | 883 | 608 | 549 | 575 | 233 | 316 | 384 | 416 | 461 |
| Cu | ppm | 366 | 271 | 285 | 34 | 22 | 327 | 606 | 322 | 62 | 23 | 22 | 388 | 321 | 306 | 355 | 520 | 402 | 376 | 158 |
| Zn | ppm | 56 | 81 | 93 | 94 | 43 | 91 | 18 | 69 | 145 | 104 | 13 | 121 | 143 | 155 | 86 | 76 | 115 | 78 | 449 |
| Ga | ppm | 6 | 4.3 | 6.8 | 5.1 | 5.6 | 7.7 | 14.2 | 3.3 | 3 | 7.6 | 14 | 5.9 | 11.6 | 13.9 | 16.7 | 13.5 | 7.4 | 11.5 | 15.8 |
| As | ppm | 5.1 | 15.8 | 9.6 | 5.8 | 81 | 0.5 | <0.5 | 83.4 | 28.5 | 25.9 | <0.5 | <0.5 | <0.5 | <0.5 | <0.5 | <0.5 | <0.5 | <0.5 | 1.2 |
| Se | ppm | 2.9 | 1.7 | 1.2 | 2.2 | <0.5 | 2.9 | 6.8 | 2.3 | 2.2 | 1 | 1.4 | 3.5 | 4.6 | 5.4 | 8.2 | 6.6 | 4.9 | 5.1 | 3.3 |
| Rb | ppm | <0.1 | <0.1 | 0.3 | 0.2 | 0.2 | 0.3 | <0.1 | 10 | 9.8 | 0.7 | 1.7 | 125.7 | 112 | 88 | 67 | 104 | 105.7 | 90.8 | 62.3 |
| Sr | ppm | 57 | 51 | 102 | 81 | 88 | 36 | 9.9 | 84.9 | 72.7 | 123.6 | 49.3 | 373.5 | 159.3 | 121 | 315 | 456 | 225.3 | 178.2 | 294.1 |
|  |  | Queluzite | |  |  |  |  |  |  |  |  | Mn-rich graphitic schist | | |  | Graphitic schist | | | | |
|  |  | **MM-9** | **MM-10** | **MM-11** | **MM-12** | **MM-14** | **MM-15** | **MM-16** | **MM-25** | **MM-26** | **MM-27** | **MM-22** | **MM-23c** | **MM-24a** | **MM-24b** | **MM-20** | **MM-21** | **MM-23a** | **MM-23b** | **MM-28** |
| Y | ppm | 12.4 | 8.9 | 14.6 | 11.4 | 9.1 | 22.5 | 43.1 | 8.4 | 10.3 | 14.1 | 31.9 | 32.4 | 31.5 | 31 | 25.2 | 33.1 | 26 | 28.7 | 22.9 |
| Zr | ppm | 55.6 | 39 | 69.7 | 50.3 | 36 | 78.9 | 137.4 | 32.1 | 35.2 | 63.1 | 117 | 91.9 | 118.3 | 95.8 | 143 | 103.2 | 93.1 | 103 | 124.5 |
| Nb | ppm | 5.2 | 4.2 | 5.7 | 5 | 4 | 5.7 | 7.7 | 3.5 | 4.7 | 6.1 | 16.2 | 6.5 | 7.3 | 5.5 | 10.2 | 6.1 | 5.8 | 5.9 | 6.7 |
| Mo | ppm | 67 | 84 | 29 | 92 | 185 | 21 | 23 | 83 | 175 | 74 | 11.2 | 35.2 | 13 | 11.2 | 14 | 15 | 9.2 | 5.2 | 1.8 |
| Ag | ppm | 0.3 | 0.2 | 0.2 | 0.4 | 0.1 | 0.4 | 0.4 | 0.1 | <0.1 | <0.1 | <0.1 | 0.2 | 0.2 | 0.2 | 0.3 | 0.2 | 0.2 | 0.2 | 0.3 |
| Cd | ppm | 12.5 | 17.9 | 0.9 | 12.1 | 10.1 | 5.7 | 0.7 | 15.6 | 18.3 | 1.2 | 0.2 | 1.7 | 2.5 | 2.8 | 0.3 | 1.8 | 2.4 | 1.2 | 1.8 |
| Sn | ppm | <1 | <1 | <1 | <1 | <1 | 1 | 2 | 1 | <1 | 1 | 3 | 2 | 2 | 2 | <1 | <1 | 2 | 2 | 6 |
| Sb | ppm | 0.2 | 0.1 | 0.2 | 0.5 | 0.8 | 0.5 | 0.4 | 0.7 | 1.1 | 2.9 | 0.2 | 0.2 | 0.2 | 0.2 | 0.1 | 0.3 | 0.2 | 0.2 | 2.6 |
| Cs | ppm | <0.1 | <0.1 | <0.1 | <0.1 | <0.1 | <0.1 | <0.1 | 0.1 | <0.1 | <0.1 | 0.1 | 6.3 | 6.2 | 4.6 | 3.9 | 2.8 | 3.8 | 4 | 2.8 |
| Ba | ppm | 6 | 3 | 6 | 10 | 6 | 2 | 17 | 723 | 624 | 51 | 39 | 4293 | 1857 | 1346 | 416 | 4645 | 1764 | 1061 | 1380 |
| La | ppm | 14.5 | 8.9 | 11.5 | 11.6 | 9.4 | 18 | 35.8 | 14.3 | 6.2 | 11.3 | 9.5 | 22.7 | 27.3 | 21.9 | 31.3 | 26 | 19.6 | 21.6 | 17 |
| Ce | ppm | 41.4 | 28.3 | 36.2 | 38.6 | 28.2 | 47.9 | 72.4 | 40.7 | 14.6 | 32.7 | 39.2 | 42.4 | 51.9 | 42.5 | 60.6 | 46.1 | 39.6 | 40.8 | 35.5 |
| Pr | ppm | 2.92 | 2.1 | 2.67 | 2.68 | 2.09 | 4.67 | 8.06 | 2.61 | 1.17 | 2.71 | 6.16 | 5.17 | 6.43 | 5.03 | 7.56 | 6.11 | 5.05 | 5.36 | 4.46 |
| Nd | ppm | 12.2 | 8.3 | 11.2 | 11.8 | 7.8 | 17.9 | 31.3 | 9.6 | 4.8 | 10.5 | 29.3 | 21.4 | 26.5 | 21.7 | 30 | 23.9 | 20.5 | 21.4 | 17.4 |
| Sm | ppm | 2.36 | 1.63 | 2.4 | 2.12 | 1.72 | 3.59 | 6.4 | 1.76 | 1.3 | 2.76 | 5.76 | 4.51 | 5.23 | 4.5 | 5.9 | 5.03 | 4.45 | 4.29 | 3.7 |
| Eu | ppm | 0.66 | 0.47 | 0.7 | 0.48 | 0.57 | 1.14 | 1.42 | 0.59 | 0.38 | 0.72 | 1.35 | 1.26 | 1.34 | 1.31 | 0.88 | 0.94 | 1.09 | 1.19 | 1.29 |
| Gd | ppm | 2.46 | 1.73 | 2.6 | 2.16 | 1.74 | 3.7 | 7.15 | 1.86 | 1.97 | 2.51 | 4.22 | 5.07 | 5.83 | 5.22 | 5.56 | 5.23 | 4.46 | 4.5 | 4.25 |
| Tb | ppm | 0.35 | 0.25 | 0.38 | 0.33 | 0.25 | 0.59 | 1.11 | 0.25 | 0.3 | 0.4 | 0.65 | 0.84 | 0.84 | 0.81 | 0.77 | 0.76 | 0.67 | 0.71 | 0.65 |
| Dy | ppm | 2.39 | 1.42 | 2.5 | 2.08 | 1.57 | 3.72 | 7.11 | 1.75 | 1.78 | 2.37 | 4.82 | 5.1 | 5.09 | 5.16 | 4.53 | 5.03 | 4.21 | 4.72 | 3.93 |
| Ho | ppm | 0.49 | 0.29 | 0.52 | 0.48 | 0.3 | 0.8 | 1.44 | 0.32 | 0.36 | 0.5 | 1.15 | 1.01 | 1.1 | 1.02 | 0.89 | 1.1 | 0.87 | 1.04 | 0.86 |
| Er | ppm | 1.33 | 0.85 | 1.52 | 1.18 | 0.89 | 2.19 | 4.42 | 0.76 | 0.98 | 1.44 | 3.84 | 3.13 | 3.15 | 3.22 | 2.55 | 3.3 | 2.68 | 3.04 | 2.43 |
| Tm | ppm | 0.19 | 0.12 | 0.2 | 0.17 | 0.12 | 0.32 | 0.58 | 0.12 | 0.14 | 0.22 | 0.55 | 0.42 | 0.46 | 0.45 | 0.33 | 0.48 | 0.34 | 0.4 | 0.32 |
| Yb | ppm | 1.27 | 0.81 | 1.34 | 1.07 | 0.75 | 2.06 | 3.77 | 0.82 | 0.97 | 1.31 | 3.68 | 2.8 | 2.94 | 2.75 | 2.08 | 3.1 | 2.4 | 2.72 | 2.2 |
| Lu | ppm | 0.17 | 0.13 | 0.22 | 0.18 | 0.11 | 0.32 | 0.61 | 0.11 | 0.13 | 0.2 | 0.59 | 0.43 | 0.46 | 0.43 | 0.32 | 0.48 | 0.36 | 0.42 | 0.32 |
| Hf | ppm | 1.5 | 1.1 | 1.8 | 1.5 | 0.9 | 2.1 | 3.4 | 0.8 | 0.8 | 1.5 | 2.9 | 2.4 | 3.0 | 2.6 | 3.9 | 2.5 | 2.4 | 2.6 | 3.1 |
| Ta | ppm | 0.3 | 0.2 | 0.2 | 0.2 | 0.2 | 0.3 | 0.5 | 0.2 | 0.3 | 0.4 | 1.1 | 0.4 | 0.6 | 0.3 | 0.4 | 0.4 | 0.4 | 0.4 | 0.5 |
| W | ppm | 43 | 30 | 6.7 | 15 | 25 | 9.9 | 4.9 | 35 | 89 | 103 | 258 | 216 | 174 | 192 | 4.6 | 5.1 | 91 | 100 | 181 |
| Au | ppb | 0.8 | 2.7 | 5.3 | 2.1 | 1.6 | <0.5 | 0.7 | <0.5 | 1.5 | <0.5 | <0.5 | 1.1 | <0.5 | <0.5 | 0.7 | <0.5 | 0.6 | <0.5 | 1.3 |
| Hg | ppm | <0.01 | <0.01 | <0.01 | <0.01 | <0.01 | <0.01 | <0.01 | <0.01 | <0.01 | <0.01 | <0.01 | <0.01 | <0.01 | <0.01 | <0.01 | <0.01 | <0.01 | <0.01 | <0.01 |
| Tl | ppm | <0.1 | <0.1 | <0.1 | <0.1 | <0.1 | <0.1 | <0.1 | 0.5 | 0.6 | <0.1 | 0.1 | 3.9 | 2.4 | 2.2 | 2.1 | 2.7 | 2.7 | 1.9 | 0.9 |
| Pb | ppm | 4.3 | 1.8 | 3.1 | 3.1 | 4.9 | 18 | 28 | 16 | 85 | 45 | 11 | 25 | 22 | 29 | 6.5 | 14 | 28 | 25 | 438 |
| Bi | ppm | 1.8 | 1.8 | 3.3 | 1.2 | 1.1 | 1.1 | 5.4 | 0.7 | 1.3 | 0.7 | 0.3 | 1.0 | 0.4 | 0.4 | 0.5 | 0.8 | 0.6 | 0.6 | 1.2 |
| Th | ppm | 3.0 | 1.9 | 2.4 | 2.0 | 1.9 | 3.8 | 6.1 | 1.9 | 0.7 | 3.0 | 11.0 | 4.4 | 5.1 | 3.8 | 5.8 | 5.5 | 4.8 | 4.6 | 2.9 |
| U | ppm | 1.2 | 0.7 | 0.7 | 0.9 | 0.6 | 2.1 | 3.4 | 0.7 | 0.3 | 1.3 | 7.6 | 3.0 | 3.3 | 2.4 | 2.4 | 3.6 | 3.1 | 3.4 | 1.2 |
|  |  |  |  |  |  |  |  |  |  |  |  |  |  |  |  |  |  |  |  |  |
